# Analytical Model to Deduce the Conformational and Dynamical Behavior in Dendrimers: A Review

**DOI:** 10.3390/polym16131918

**Published:** 2024-07-05

**Authors:** Shelly Bhardwaj, Amit Kumar

**Affiliations:** Theory & Simulation Laboratory, Department of Chemistry, Jamia Millia Islamia (A Central University), New Delhi 110025, India

**Keywords:** conformation, dendrimer, excluded-volume, relaxation, rheology, semiflexibility

## Abstract

This review utilizes an optimized Rouse–Zimm discrete hydrodynamic model and the preaveraged Oseen tensor, which accurately consider hydrodynamic interactions to study model dendrimers. We report the analytical theories that have been previously developed for the creation of generalized analytical models for dendrimers. These generalized theories were used to assess the conformational and dynamical behavior of the dendrimers. By including stiffness in the bonds, the neglect of excluded volume interactions may be somewhat offset. This is true at least in the case of short spacers. While the topological limitations on the directions and orientations of the individual bond vectors in dendrimers implement semiflexibility, the intensity of these contacts was determined by the potential geometric orientations of the bonds, and later on the excluded volume interactions in dendrimers, which were described in terms of the effective co-volume between nearest non-bonded monomers and modeled using the delta function pseudopotential. With the aid of the models developed, the authors condensed various conformational and dynamic properties of dendrimers that depend on their degree of semiflexibility and the strength of the excluded volume. These analyses came to the conclusion that the flexible dendrimer in one limit and the earlier described freely rotating model of dendrimers in the other constitute a highly generalized way of capturing a wide range of conformations in the developed mathematical model in dendrimers.

## 1. Introduction

**Figure 1:**
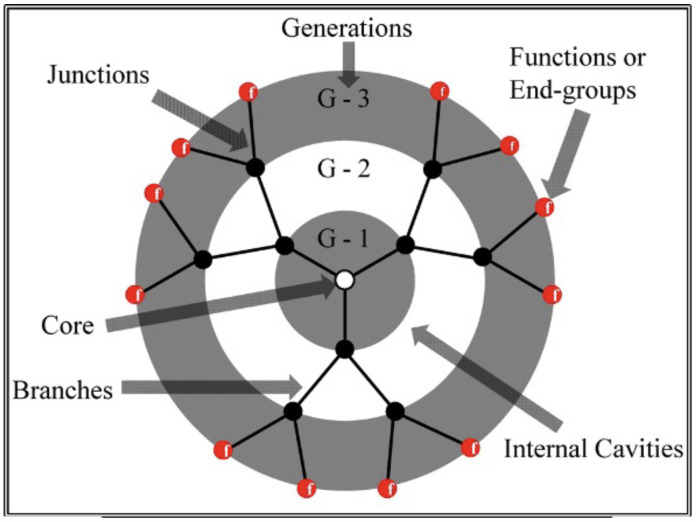
A schematic diagram illustrating the topological structure of dendrimers is presented, wherein the total number of monomers may be determined using the formula $N=1+Pf\frac{{\left(f-1\right)}^{G}-1}{f-2}$ in which *f* = functionality, *P* = spacers and *G* = generation of dendrimers.

The ideal Cayley tree-like macromolecules called [1] dendrimers, first introduced by Donald A. Tomalia [2], are symmetrical, highly branched, monodispersed, acyclic polymers that are built around a central multipurpose core. According to topological theory, dendrimers are made up of a central core with increasing monomer density, which is denoted by functionality *f*, and a number of concentric shells, which are shown by the branch points that spread out from the core [3]. Discrepancies in morphology with linear polymers are evident in their physicochemical characteristics [4]. This precision in dendrimer synthesis is what distinguishes them from traditional polymers and imparts a remarkable level of control over their properties and functions. Because of the various properties and applications associated with dendrimers, there is a crucial requirement for methods to characterize them. However, this undertaking is inherently challenging due to the unique nature of their structure [5]. Figure 1 shows that the spacers “*P*” are the linear segments connecting the successive branch points, with the number of generations, denoted as “*G*”, being dictated by the number of spacers between the central core of the dendrimer and each endpoint.

The branched shell specifies the type and volume of interior void space that are encircled by the terminal groups as a function of the number of growth generations, whereas the core can be viewed as the molecular information hub from which the size, shape, directionality, and number of branches are expressed through covalent connections to the outer shells.

They have a variety of special qualities because of their structure [4,6] including significant penetrating power and a lot of functionalized terminal groups. The well-defined dendrimer architectures, with their precisely tunable size, shape, and branching patterns, offer an ideal platform for testing the accuracy of analytical models and computational simulations. This characteristic enables researchers to gain deeper insights into the behavior of macromolecules in various environments. Furthermore, dendrimer’s monodispersity ensures that each molecule within a given dendrimer generation is nearly identical in size and structure. This uniformity simplifies experimental measurements and enhances the reliability of comparative studies. In the past decades, theoreticians have confidently investigated the conformational dynamics, rheological behavior, and other properties of dendrimers, knowing that variations due to molecular heterogeneity are minimized. As a result, dendrimers have become valuable tools for validating and refining theoretical predictions, enhancing the robustness and applicability of models and simulations in fields such as polymer science, soft matter physics, and materials engineering.

In this review, we report the development of such comprehensive theories for deducing the conformational and rheological behavior of model dendrimers in diluted solutions using the optimized discrete Rouse–Zimm hydrodynamic model with the preaveraged Oseen tensor widely incorporating the hydrodynamic interactions. We appraised the conformational and dynamical nature of model dendrimers accessed through different analytical techniques by the authors of the respective articles. These features were influenced by the degree of semiflexibility and the parameters of excluded volume interactions. The text also includes summaries and an inquiry into prospective areas for further research.

## 2. Analytical Theories and Development of Analytical Models for Dendrimers

Understanding analytical models of dendrimers is crucial due to their intricate structures and versatile applications. These models provide predictive power, enabling the exploration of dendrimer behaviors under various conditions, thus saving time and resources in the development process. They offer insights into dynamic conformational changes, aiding in the optimization of dendrimer properties for specific applications. Additionally, analytical models contribute to a fundamental understanding of dendrimer behavior and allow for systematic comparisons, ensuring the reliability of experimental findings [7]. Furthermore, the conformational or dynamical properties obtained from analytical models are verified through the use of molecular dynamic simulations [8].

To implement these advantages, the behavior is studied through the Rouse–Zimm model [9], which is a fundamental concept in polymer physics, offering crucial insights into the dynamic characteristics of polymer chains in solution. These models simplifies the representation of polymers as a series of interconnected segments or beads, each undergoing Brownian motion independently. What sets this optimized Rouse–Zimm model apart is its incorporation of hydrodynamic interactions between these segments, considering how a moving segment drags along its neighbors due to solvent resistance, which was initially not included in the work of P. E. Rouse [10]. This dynamic coupling effect is central to understanding polymer behavior. The model is particularly influential in explaining the rheological properties of polymer solutions, elucidating the relationship between concentration, molecular weight, and viscosity. It also provides a framework for studying conformational changes in polymer chains under different conditions, shedding light on phenomena such as stretching, coiling, and relaxation. The optimized Rouse–Zimm [9] discrete hydrodynamic model, which simulates the dendrimer in the solvent, is now the basic theoretical framework used to predict these properties while neglecting the long-range excluded volume interactions.

Since the choice of solvent has a considerable impact on the behavior and properties of complex macromolecules, the function of solvents in understanding the model, structure, and dynamics of dendrimers is of utmost relevance, since they have an impact on their conformation, solubility, and interactions. Their solubility is a key factor in how solvents affect dendrimer dynamics [7]. Due to their highly branched and frequently hydrophobic architectures, dendrimers can display a variety of solubility traits depending on the polarity, interactions of the solvent chemical nature of dendrimers, solvent–solute interactions, solvent polarity, etc. Dendrimer dynamics in good and bad solvents are of great interest [11]. The qualities of a “good solvent” for dendrimers promote expansion and solvation. Dendrimer conformation is stretched due to significant solvation forces from these solvent’s attraction for the structure. They swell and move more under such situations. A “poor solvent”, however, may not be good for dendrimer solvation. Dendrimer–solvent interactions are weaker, reducing solvation forces and compacting the dendrimer. Poor solvents may restrict dendrimer movement and promote aggregation [12,13]. Therefore, “solvents influence dendrimer dynamics” is highlighted. The rate of dendrimer diffusion, conformational changes, and molecular interactions can all be impacted by the solvent’s viscosity and polarity. Reduced intermolecular interactions among the sparsely distributed dendrimers in diluted fluids makes it possible to isolate and study their intrinsic dynamics. This separation is especially useful for comparing experimental results with analytical models, such the Rouse–Zimm model [9], which frequently applies best under diluted settings.

Several experimental techniques, including nuclear magnetic resonance (NMR) spectroscopy, light scattering, and single-molecule tracking perform well in diluted solutions and provide great precision and sensitivity for characterizing dendrimer dynamics. Furthermore, the study of fundamental dendrimer characteristics, such as conformational dynamics, segmental mobility, and reactions to environmental stimuli, is best conducted in diluted solutions. Additionally, they help researchers investigate how various solvents affect dendrimer behavior, adding to our understanding of how dendrimer–solvent interactions function [14]. Hence, these calculations hold true when considering spacers of significant length, owing to the concept of “solvents” and the assumption that the dendrimer solution is extensively diluted.

In macromolecular studies, it is customary to analytically depict the macromolecular chains as a group of *N* beads, which can be either simple monomer units or effective monomer units. These beads are assigned coordinates $R_{i}$. The theoretical representation of the macromolecular chains involves a series of stochastic Langevin equations for each individual bead up until the point where a coarse-grained model is employed. In relation to certain dendrimers that have been recently synthesized, specifically those with comparatively short spacers, significant excluded volume interactions are anticipated, rendering the conventional model unsuitable [9,15]. Excluded volume interactions manifest when the branches of dendrimers are in such close proximity that they are unable to coexist inside the same spatial region simultaneously. The substantial nature of these interactions poses challenges in the application of conventional approaches. Based on the present scenario, the approach to tackle this particular matter involves approximating the behavior of short-spacer dendrimers by the imposition of precise topological constraints governing the movement and orientation of the dendrimer’s branches. The study’s model, developed by the Biswas group [16,17,18,19,20], restricts the possible directions and orientations of the constituent bond vectors. Using normalized spherical harmonics, the restrictions placed on the orientations of individual bonds are stated. Also, it could be possible to explain the omission of excluded volume interactions by incorporating a small amount of semiflexibility into the optimal Rouse–Zimm technique. These models view dendrimers as discontinuous semiflexible polymers, in contrast to continuum chain models, such as the worm-like chain [21], which sees polymers as continuous, smooth curves driven by differential equations. The branching, stiffness, and hierarchical organization of dendrimers are better represented by the discrete representation of dendrimers. Furthermore, in context of studying the behavior of dendrimers in a dilute solution, the incorporation of hydrodynamic interactions becomes imperative [22]. The Oseen tensor can be used mathematically to depict the hydrodynamic interactions, or **[H·I]**, between the *i*-th and *j*-th monomers. These interactions are affected by the solvent [23]. To embrace all these parameters, the Langevin equation offers a theoretical foundation for comprehending the dynamics of these macromolecules. This equation elucidates the dynamics of a particle, polymer chain, or dendrimer as it navigates through a dynamic environment, accounting for stochastic forces and the influence of friction and also with external stimuli [24]. The discrete representation of semiflexible polymers allows for the use of eigenvectors and eigenvalues in solving the matrix representation of the Langevin equation. By utilizing the concepts of eigenvectors and eigenvalues, it is possible to perform matrix diagonalization, hence reducing the system of equations. The process of diagonalization provides insight into the characteristic modes or patterns of motion exhibited by the polymer chain [23,25,26]. Every eigenvalue is connected with a certain mode of motion, whereas the accompanying eigenvector characterizes the spatial pattern related to that particular mode [27].

The Langevin equation of motion undergoes simultaneous curvature as it alters the spatial coordinates of atoms within a macromolecular structure in the optimized Rouse–Zimm formalism skeleton, which is in line with the progression of time [9]. The ignoring of the inertial term of the individual units is justified in the study of dendrimer dynamics due to their overdamped nature [23]. The inclusion of damping would result in a decrease in the effective frequency. The fundamental Langevin equation that determines the spatial position of the *i*-th monomer, represented by the vector $R_{i}\left(t\right)$, can be expressed as follows:

(1) $$\zeta \frac{\partial R_{i}\left(t\right)}{\partial t}=-\frac{3k_{B}T}{l^{2}}\sum_{j=1}^{N}{\left[H\cdot A\right]}_{ij}R_{j}\left(t\right)+f_{i}\left(t\right)$$

The summation of intramolecular force, $3k_{B}T/l^{2}\sum_{j=1}^{N}{\left[H\cdot A\right]}_{ij}R_{j}\left(t\right)$, and random force, $f_{i}\left(t\right)$, cancels out the viscous force, $\zeta \partial R_{i}\left(t\right)/\partial t$, on the left-hand side of Equation (1). The random force, represented by $f_{i}\left(t\right)$, is assumed to be distributed according to a Gaussian white noise model with a zero mean ($\langle f_{i}\left(t\right)\rangle =0$). Additionally, it is assumed to have delta function correlation, given by $\langle f\left(t\right)\cdot f\left(t^{\prime }\right)\rangle =2k_{B}T\zeta \delta \left(t-t^{\prime }\right)$, where $k_{B}$ represents the Boltzmann constant.

The process for solving the Langevin equation, Equation (1), entails the following sequential phases. The problem can be resolved by separating the position coordinates $R_{i}\left(t\right)$ of monomers into distinct normal modes, which are denoted as X. The diagonalization of the matrix product $\left[H\cdot A\right]$ can be achieved using matrix diagonalization techniques [16]. The normal coordinates, represented as X, are obtained by transforming the monomer coordinates, which are denoted as R.

(2) $$R_{j}\left(t\right)=\sum_{k=1}^{N}Q_{jk}\cdot X_{k}\left(t\right)$$

The matrix Q is constructed using a set of linearly independent eigenvectors that have been normalized. These eigenvectors are derived from the matrix product $\left[H\cdot A\right]$. The expression $Q^{-1}{\left[H\cdot A\right]}_{ij}Q=\Lambda$ defines the variable Λ. The members of the diagonal matrix Λ are denoted as $\lambda_{k}$. The eigenvectors, denoted as Q, can also be employed to derive a diagonal matrix, denoted as γ, where the elements represent $\mu_{k}$ (as the mean-square length). The individual diagonalization of the connection matrix is denoted as $\left[A\right].$ The calculation of γ through the eigenvectors Q is expressed as $Q^{T}{\left[A\right]}_{ij}Q=\gamma .$ Therefore, the Langevin equation derived in relation to generic normal coordinates is Equation (3) [28]. By averaging the squares of independent normal modes, these values give the mean-square length of the *k*-th normal mode, which is given by the formula $\langle X_{k}^{2}\rangle =l^{2}\mu_{k}^{-1}$ (where $\mu_{k}=1/\lambda_{k}$).

(3) $$\zeta \frac{\partial X_{k}\left(t\right)}{\partial t}=-K\lambda_{k}X_{k}\left(t\right)+f_{k}\left(t\right)$$

and correspondingly, the relaxation rate of normal coordinate $X_{k}$ is given by $\langle X_{k}\left(t\right)\cdot X_{m}\left(0\right)\rangle =\delta_{km}\langle X_{k}^{2}\rangle \exp\left(-tK\lambda_{k}/\zeta \right)$.

### 2.1. Dendrimers with Semiflexibility

In the context of linear chains, the concept of stiffness is established by ensuring a consistent average angle between successive bond vectors. The aforementioned methodology is expanded to encompass dendrimer models of various complexity with the incorporation of limitations on the orientations of bond vectors [29,30,31,32]. Previously, Dolgushev and Blumen [32] used an adaptation of the Rouse model that included bond orientation limitations to introduce semiflexibility. Semiflexibility was achieved by constraining bond orientations using the Bixon–Zwanzig (BZ) method. The stiffness degree (SD) was implemented using a matrix *W* formed from bond orientation restrictions and an inverse matrix *V*. The parameters for flexibility and semiflexibility were employed. The SD varies from completely flexible (t=0) to semiflexible (t>0). The stiffness parameter *q* was employed, particularly for dendrimers, with values as low as q=0.4. A further study [33] studied the dynamical properties of chain polymers and dendrimers with junctions exhibiting varied degrees of stiffness (SD). It focused on how stiffness differences affect the macroscopic behavior of polymers.

**Figure 2:**
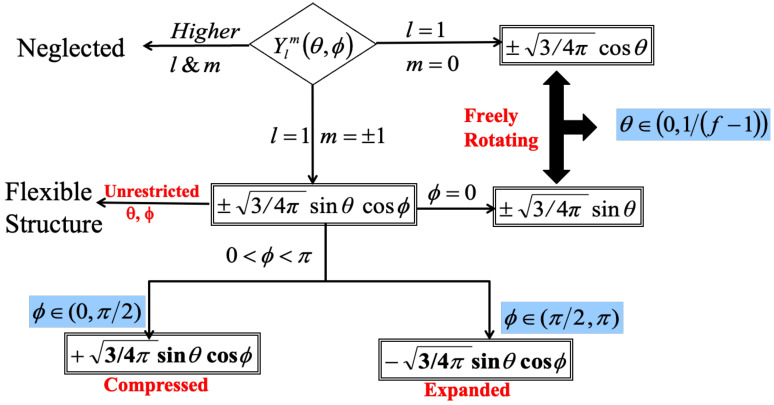
A visual depiction in the form of a flowchart is presented, illustrating the various values of the directional angle (θ) and the orientational angle (ϕ). These angles are constrained to the range of 0≤ϕ≤π. The flowchart employs spherical harmonics to depict these values [16,17].

In another analytical model, the orientation restrictions were represented by normalized spherical harmonics $Y_{l}^{m}\left(\theta ,\phi \right)$, where $Y_{l}^{-m}$ denotes the complex conjugate of $Y_{l}^{m}$, as proposed by the authors [16,17]. The flowchart depicted in Figure 2 provides a visual representation of the directional angle (θ) and orientational angle (ϕ) values. The range of ϕ is defined as 0≤ϕ≤π. The visualization of these angles is achieved by the utilization of spherical harmonics. The utilization of this specific analytical model leads to the matrix representation of the Langevin equation, which may be solved by employing eigenvectors and eigenvalues [16,17].

The utilization of normalized spherical harmonics $Y_{l}^{m}\left(\theta ,\phi \right)$ was employed inside a spherical polar coordinate system to represent bond orientation limitations. This function facilitates the consideration of interdependencies among successive bonds, encompassing the impact of adjacent bonds. The initial approach involved estimating the correlation by imposing a constraint on the angle θ between bond vectors. Nevertheless, actual dendrimers impose constraints on rotational movement, thereby extending this correlation across many bond orientations. The establishment of a mutual reliance necessitates the accurate determination of bond orientation, which is quantified by the angle ϕ as stated by Kumar and Biswas [16]. Equation (4a) represents the average scalar product of adjacent bond vectors within a non-symmetric rotating potential. In a similar manner, the shortest topological distance between non-adjacent bonds denoted by indices *i* and *k* is determined according to Equation (4b).

(4a) $$\langle l_{i}\cdot l_{j}\rangle =\pm l^{2}Y_{l}^{m}\left(\theta ,\phi \right)$$

(4b) $$\langle l_{i}\cdot l_{k}\rangle =\langle l_{i}\cdot l_{j_{1}}\rangle \cdot \langle l_{j_{1}}\cdot l_{j_{2}}\rangle \ldots \langle l_{j_{n}}\cdot l_{k}\rangle \frac{1}{l^{2n}}$$

The calculation of the average scalar product of bond vectors was performed [16], which is accompanied by the comprehensive analysis of the angular dependence of bond vectors expressed in terms of spherical harmonics:

(5) $$\frac{\langle l_{i}\cdot l_{j}\rangle }{l^{2}}=\sqrt{\frac{3}{4\pi }}\sin\theta \cos\phi$$

Therefore, by adjusting the bond projection angle, ϕ, the relative orientation of bond vectors can be changed. A compressed conformation was produced by a small ϕ value, whereas an extended conformation was produced by a big ϕ value. While in contrast to La Ferla’s work [30], the bond angle for two vectors with a head-to-tail orientation was calculated using the formula arccos (−p) for the cosine of the angle between consecutive bond vectors, which is denoted as “*p*”. The average bond vector scalar product was thus represented by Equation (6) in terms of the local stiffness parameter $p_{k}$

(6) $$\frac{\langle l_{i}\cdot l_{j}\rangle }{l^{2}}=\prod_{k=1}^{D_{ij}}p_{k}$$

**Figure 3:**
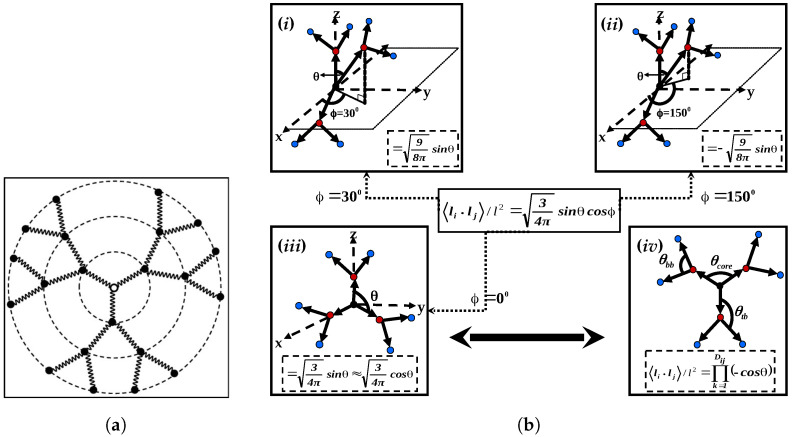
(**a**) Simple bead-spring model dendrimers are shown schematically along with four different types of generated conformations in semiflexible dendrimers: (**b**-(**i**)) compressed conformations, (**b**-(**ii**)) expanded conformations, (**b**-(**iii**)) freely rotating conformations, and (**b**-(**iv**)) La Ferla’s model dendrimers [30]. Reprinted with permission from [16], copyright 2010 American Chemical Society, and reprinted from [17] with the permission of AIP Publishing.

Amidst these concurrent considerations regarding bond orientations, a dendrimer’s structural arrangement is illustrated as a model in Figure 3 along with a schematic illustration that clarifies the direction and orientation angles between bond vectors [16]. Generally, the three different types of angles are (A) $\theta_{core}$, which represents the relationship between any two bonds within the dendrimer’s central core; (B) $\theta_{tb}$, which describes the angle formed between a bond vector and the primary branch; and (C) $\theta_{bb}$, which represents the relationship between two bond vectors within a branch that originates from the same monomer. The equilibrium scalar product of the bond vectors is used to create a bond–bond correlation matrix based on the corresponding spherical harmonics computed for each pairing of θ and ϕ [16]. The model representation in Figure 3 gives a foundation for comparison. The dendrimer model, which incorporates the idea of semiflexibility, is illustrated in part of the comparison of analytical models in Figure 3 by graphs (b-(i)) and (b-(ii)), which exhibit compressed and expanded conformations, respectively. Contrarily, graph (b-(iii)) depicts a conformation where bond orientation is unrestrained (ϕ=0), which is similar to Ferla’s freely rotating model [30] that disregards orientational angles. In contrast to a model that assumes unlimited bond orientation, this comparison in the study of author [16] helps to clarify the impact of semiflexibility on dendrimer conformations.

### 2.2. Dendrimers with Excluded Volume

As discussed in earlier parts, an improved Rouse–Zimm [9] technique is used to look at the dynamic properties of dendrimers in dilute solutions. Semiflexibility is achieved by incorporating the topological limitations imposed on the orientations and alignments of the associated bond vectors through replication. This choice of modeling allows excluded volume interactions to be left out, which is helpful when working with dendrimers with short spacers. Even though these methods can predict the rheological properties in a general way, ignoring the missing volume interactions has been seen as a major flaw. They have a big effect on how dendrimers are shaped, which makes dendrimers change their structures to avoid bumping into other molecules. Also, removed volume interactions have a big effect on how and where dendrimers move around in the solvent. Because there are so many interactions going on inside the molecule, it can be hard to analyse deleted volume interactions. Scaling and a method called “renormalization group analysis” were used to figure out how big starburst [34,35] dendrimers were in the early attempts to understand this. In another study, researchers assess these interactions by finding a balance in the molecule’s own energy using a method called self-consistent minimization [36,37].

**Figure 4:**
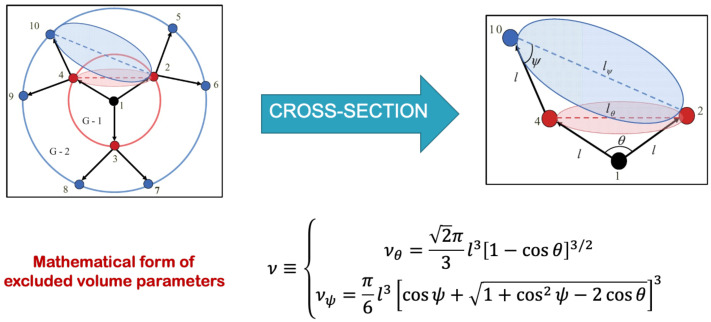
The closest non-bonded connections between monomers from the same generation and generations before it in the model dendrimer. Reprinted from [38] with the permission of AIP Publishing.

In order to tackle this issue, the authors [38] proposed a novel methodology for integrating the excluded volume interactions between two non-bonded monomers into a dendrimer structure. The methodology under consideration takes into account the structural geometry and differentiates between two distinct categories of excluded volume interaction parameters, which are specifically denoted as $v_{\theta }$ and $v_{\psi }$. These interactions pertain to the excluded volume phenomenon observed between non-bonded monomers situated within the same generation of the dendrimer as well as those in the immediately adjacent generation. The determination of these excluded volume interactions was dependent on the structural topology of the dendrimer. It is important to highlight that the modeling technique employed in this study [38] focused on accurately representing the short-range non-bonded interactions between monomers. As a result, this approach is particularly well suited for densely packed molecular structures such as dendrimers (see Figure 4). Nevertheless, the model fails to account for the impact of excluded volume interactions observed in elongated linear polymers.

Hence, in the process of solving the Langevin equation for dendrimers that incorporate excluded volume [38], it is possible to break down the interaction potential $U\left(\{{\vec{R}}_{p}\}\right)$ into two distinct constituents. The first component encompasses the bonded potential, which takes into consideration the interactions between monomers that are directly linked by a bond. On the other hand, the second component is the non-bonded interaction potential, which emerges as a consequence of the excluded volume effects [38].

(7) $$\frac{U\left(\{{\vec{R}}_{p}\}\right)}{k_{B}T}=\frac{3}{2l^{2}}\sum_{i,j}A_{i,j}{\vec{R}}_{i}{\vec{R}}_{j}+\frac{1}{2l^{2}}\sum_{i,j}v_{i,j}E_{i,j}\delta \left(R_{ij}\right){\vec{R}}_{i}{\vec{R}}_{j}=\frac{1}{2l^{2}}\sum_{i,j}A_{i,j}^{ex}{\vec{R}}_{i}{\vec{R}}_{j}$$

In the theoretical model studied by the authors [38], it was postulated that all bonds present within the dendrimer possess an equivalent spring constant, which is represented as $K=3k_{BT}/l^{2}$. Here, $k_{B}$, *T*, and $l^{2}$ correspond to the Boltzmann constant, temperature, and mean square length of the unstretched spring, respectively. The matrix denoted as A in Equation (7) represents the connectivity of monomers through bonds, and it possesses the property of symmetry. The spatial arrangement of the nearest non-bonded monomers was represented by the square matrix E of size N×N. $A^{ex}=\left(3A+vE\right)$ in Equation (7) is the N×N square matrix, which accounts for not only the connectivity of monomers in the dendrimer but also the presence non-bonded interactions.

The excluded volume parameters, denoted as $v_{ij}$ in Equation (7), quantify the intensity of excluded volume interactions between non-bonded monomers *i* and *j*. These interactions can be characterized as either $v_{\theta }$ or $v_{\psi }$, depending on whether the monomers belong to the same generation or adjacent generations, respectively.

The precise geometric method for calculating these excluded volume interaction parameters has been explained in the research undertaken by Biswas et al. [38]. The monomers in the bead-spring model are represented as spherical objects. The diameter of these spheres, labeled as $l_{\theta }$ and $l_{\psi }$ as in Figure 4, was determined using the cosine formula. It was assumed that the side lengths of the spheres are equal. The resulting equation for $l_{\theta }$ is $l_{\theta }=l{\left(1-\cos_{\theta }\right)}^{1/2}$, where θ is derived from the Δ 1, 2, 4. Similarly, they calculated the length $l_{\psi }$ using the cosine formula based on the triangle Δ 2, 4, 10, assuming that the angles 4, 10, 2 represent $l_{\psi }$. The volume of the sphere $v_{\theta }$ represents the excluded volumes between the nearest non-bonded monomers in the same dendrimer generation. Similarly, the volume of the sphere $v_{\psi }$ denotes the excluded volumes between monomers in neighbouring dendrimer generations. These volume parameters are described by Equation (8).

(8) $$v_{\theta }=\frac{\sqrt{2\pi }}{3}l^{3}{\left[1-cos\theta \right]}^{3/2}\;\;\;\&\;\;\;v_{\psi }=\frac{\pi }{6}l^{3}{\left[cos\psi +\sqrt{1+cos^{2}\psi -2cos\theta }\right]}^{3}$$

It is important to keep in mind that this method works best when there are no charged monomers, and long-range interactions are thought to be short-range and insignificant past a certain distance between interacting monomers. Using the interaction potential, $U\left(\{{\vec{R}}_{p}\}\right)$, the Langevin equation (see Equation (1)) can be written in a simpler way as Equation (9), which captures the dynamics of the system within the modeling framework.

(9) $$\zeta \frac{\partial {\vec{R}}_{i}\left(t\right)}{\partial t}=\frac{K}{3}\sum_{j=1}^{N}{\left(H\cdot A^{ex}\right)}_{ij}{\vec{R}}_{j}\left(t\right)+f_{i}\left(t\right)$$

## 3. Directly Observable Quantities

When investigating the dynamics of dendrimers using the Langevin equation, various directly measurable parameters offer useful insights into the behavior and properties of these intricate macromolecules. The comprehension of the dendrimer’s behavior in many applications, including medication delivery [39], materials science [40], and nanotechnology [41], necessitates a thorough grasp of their fundamental structural and dynamical features [42]. The measurement of intramolecular distances pertains to the determination of the spatial separation between particular spots or groups inside the dendrimer structure, hence providing insights into the local structural characteristics [43]. Conformational flexibility refers to the dendrimer’s capacity to assume many conformations, which in turn influences its interactions with other molecules. Dynamical properties encompass various characteristics of dendrimers, one of which is the diffusion coefficient (*D*). This coefficient serves as a measure of the speed at which dendrimers traverse a solvent, providing valuable insights into their mobility. The intrinsic viscosity, denoted as [η], is a parameter that quantifies the resistance of a dendrimer to flow within a solvent. It provides insight into the dendrimer’s size and form characteristics. The orientational relaxation time is a measure of the duration required for dendrimers to undergo reorientation within a solvent. The concept of fractal dimensions is employed to characterize the self-similar or fractal properties exhibited by dendrimer formations. The relaxation moduli $G^{\prime }\left(\omega \right)$ and $G^{\prime \prime }\left(\omega \right)$ characterize the elastic and viscous behaviors shown by dendrimers when subjected to external forces over varying temporal regimes.

### 3.1. Rheological Nature of Model Dendrimers

Rheological research is utilized to explore the influence of macromolecules on the mechanical response of materials under external stresses. The mechanical and flow properties of materials, particularly in relation to dendrimers, provide significant insights into their impact on many systems. Rheological studies in dendrimers encompass various significant elements and applications, including viscosity modification, viscoelasticity, and flow behavior analysis. The eigenvalues and normalized eigenvectors of matrices play a significant role in the computation of dynamic features of dendrimers, as discussed previously. Intrinsic viscosity is dictated by the eigenvalues, whereas the translational diffusion coefficient is affected by both the eigenvalues and the normalized eigenvectors of the matrix product of the hydrodynamic interaction matrix and the connection matrix, which is denoted as [H·A]. In contrast to star or linear polymers, dendrimers demonstrate distinctive characteristics with regard to their intrinsic viscosity. Rather than exhibiting a continuous increase throughout generations, the inherent viscosity of dendrimers achieves its maximum value at a specific generation, which is referred to as the peak or maximum. This behavior has also been documented in empirical research [7,16,44] where they provided a qualitative explanation for the observed behavior of dendrimers. The primary factor contributing to this behavior is from the relationship between the mass or molecular weight of dendrimers and their production.

The mass or molecular weight of dendrimers exhibits an exponential increase as their creation progresses. Nevertheless, the pace at which their volume increases is not proportional; rather, it adheres to a cubic power-law pattern. This implies that until a specific generation is reached, the rate at which the volume of the dendrimer grows exceeds the rate at which its molecular weight increases. As a result, there is a positive correlation between the growth of the dendrimer and the intrinsic viscosity [η], indicating that as the generation of the dendrimer grows, so does its intrinsic viscosity. After reaching the maximum, there is a further reduction in the inherent viscosity values [η]. The precise generation at which this maximum is observed is contingent upon the dendrimer’s topology, which refers to its specific structural arrangement. The manifestation of this behavior may vary between various generations of dendrimers owing to their distinct shapes and sizes. As previously stated, a typical trend in the behavior of intrinsic viscosity has been identified for various dendrimer models.

Mansfield and Klushin [45] studied the hydrodynamic radii of poly(amido amide) (PAMAM) starburst dendrimers using intrinsic viscosity measurements. It compares experimental PAMAM radii with theoretical models, particularly the Lescanec–Muthukumar configurational model [46]. The study discovers that hydrodynamic radii rapidly grow with increasing generation number, indicating a structure with stretched tiers and a hollow core. Lescanec–Muthukumar’s [46] simulations indicate a more folded structure with higher density toward the core. Using Zimm and Fixman’s intrinsic viscosity formulas [47], computer simulations were utilized to calculate the hydrodynamic radii ($R_{h}$), radius of gyration ($R_{g}$), and radius $R_{90}$. The study found that $R_{h}$ increases faster with each generation than $R_{g}$ or $R_{90}$, indicating considerable densification as generations increase. The computed radii of Lescanec–Muthukumar [46] starbursts with stiff spacers were consistent with experimental PAMAM radii. So, the primary takeaway is that measuring the hydrodynamic radius using inherent viscosity might be misleading if taken too literally. Mourey et al. [44] studied the intrinsic viscosity of polyether dendrimers utilizing size exclusion chromatography (SEC) and molecular weight-sensitive detection. They identified dendrimers from the 0–4th generation with narrow chromatogram distributions and negligible widening. With increasing generation, the intrinsic viscosity reached a maximum, whilst the refractive index increased to a minimum. Hydrodynamic radii grew almost linearly with generation. These findings coincided with the Lescanec–Muthukumar model [46], showing a decreasing density profile from the center outward. The study revealed polyether dendrimer’s distinct behavior when compared to linear polymers, demonstrating a clear maximum in intrinsic viscosity and predictable variations in density distribution during generation. Ganazzoli et al. [36] used self-consistent free-energy minimization to study the structural characteristics and intrinsic viscosity of dendrimers under excluded-volume conditions. This approach gives information about both local conformation and overall molecular dynamics. The study derived the radius of gyration ($R_{g}$) and viscometric radius ($R_{\eta }$) from intrinsic viscosity. The results revealed that intramolecular swelling is localized in the core with dendrons expanding outward and little inter-dendron mixing. Universal variables for characteristic radii resulted in a master curve for any generation or solvent quality. Molecular expansion is most noticeable in the core, and intrinsic viscosity rose progressively with generation number without reaching a maximum under ideal solvent conditions. The predicted radii and inherent viscosities were consistent with experimental and simulation data, demonstrating the model’s correctness. Drew and Adolf [48] studied the intrinsic viscosity of trifunctional dendrimers by equilibrium molecular dynamics simulations in an explicit Lennard–Jones solvent. They calculated the intrinsic viscosity of dendrimers from generations 3–7, revealing a peak at generation 5. This result is consistent with the experimental evidence and earlier stochastic simulations. For this, two methods were used: the standard Green–Kubo approach and a direct calculation method, both of which produced consistent findings. In contrast, the Flory–Fox approximation [49] exhibited a declining tendency. The investigation confirmed that intrinsic viscosity peaks at generation five before falling, as opposed to linear polymers, which show a monotonic increase. The findings highlight the importance of structure and density distribution in determining intrinsic viscosity. Monte Carlo simulations were used to determine the intrinsic viscosity of polyamidoamine (PAMAM), polypropylene-imine (PPI), and polybenzylether (PBzE) dendrimers [50]. The study’s goal was to reproduce intrinsic viscosity over generations and better understand experimental differences. The results revealed a maximum inherent viscosity as a function of generation number, which is governed by friction radius and structural features. The models for PAMAM, PPI, and PBzE were consistent with experimental results for radius of gyration and inherent viscosity. PAMAM-EDA’s intrinsic viscosity in water peaked at generation 5. The study discovered that the frictional radius influences both the intrinsic viscosity value and the location of its maximum, stressing the need to precisely model hydrodynamic interactions. The simulation method for determining dendrimer intrinsic viscosity has been improved [51]. The work used Monte Carlo simulations and the Fixman variational technique to produce numerous major results. First, adding a correction term for individual friction beads increased the accuracy of intrinsic viscosity estimations for PPI dendrimers in water. Furthermore, the use of realistic angle distributions improved the consistency between simulation findings and experimental data for PBzE monodendrons and tridendrons in THF. The distance distribution generated from MD simulations with explicit solvent was a better fit to experimental data for both the radius of gyration and intrinsic viscosity of PBzE dendrimers. The improved model effectively recreated the experimental intrinsic viscosity maximum for tridendrons, demonstrating the value of rich structural information in simulations.

**Figure 5:**
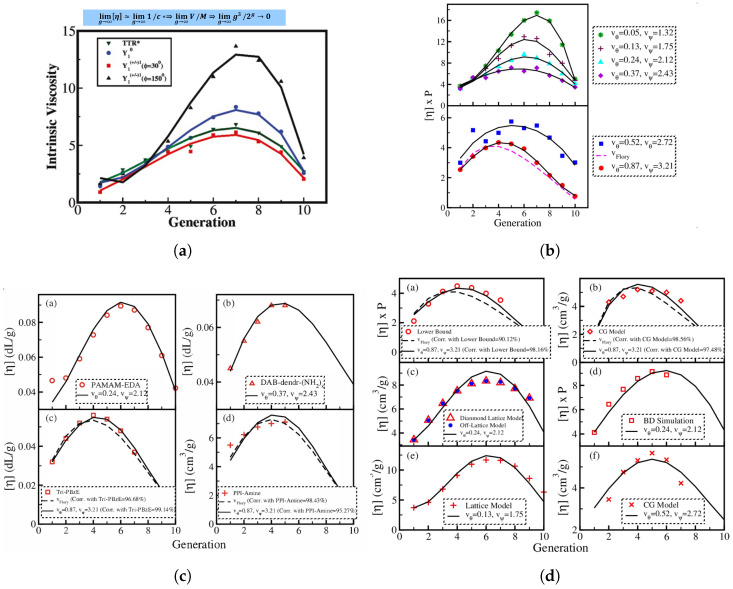
Graph (**a**) represents the comparison between analytical models of Kumar and Ferla [16]. Graph (**b**) represents the comparison between analytical models of Rai and Flory [52]. Graph (**c**) represents the comparison between analytical model and experimental results. Graph (**d**) represents the comparison between analytical model and simulation results for the determination of qualitative behavior of intrinsic viscosity. (**a**) Reprinted with the permission from [16], copyright2010 AmericanChemicalSociety. (**b**) Reprinted from [52] with the permission of AIP Publishing. (**c**) Reprinted from [52] with the permission of AIP Publishing. (**d**) Reprinted from [52] with the permission of AIP Publishing.

The researchers [16] conducted a comparative analysis between their findings on the intrinsic viscosity of semiflexible dendrimers, namely those with bond orientation angles of $\phi =30^{\circ }$, $\phi =150^{\circ }$, and $\phi =0^{\circ }$. This comparison was made with La Ferla’s TTR model, which offers an alternative method for incorporating stiffness in dendrimers. A resemblance was noted in the qualitative behavior displayed in Figure 5a. All models exhibited a distinct peak in intrinsic viscosity at a certain generation, although the precise location of these peaks differed across the different models. In the scenario when $\phi =0^{\circ }$, the intrinsic viscosity pattern exhibited by Kumar’s model [16] bears a striking resemblance to that of La Ferla’s TTR model [30], including a comparable range. Nevertheless, the numerical values of intrinsic viscosity [η] exhibited a little elevation compared to the comparable values obtained from the La Ferla model. Transitioning to the compressed semiflexible dendrimers characterized by a bond orientation angle of $\phi =30^{\circ }$, it was observed that their intrinsic viscosity exhibits comparable patterns and magnitudes to those of the TTR model [30]. In contrast, the enlarged dendrimers with a bond orientation angle of $\phi =150^{\circ }$ exhibited a wider range of intrinsic viscosity values, notably in the vicinity of the peak, while maintaining a qualitatively similar overall shape. Dendrimers exhibiting a bond orientation angle (ϕ) of $0^{\circ }$ occupy an intermediate position in relation to the expanded and compressed conformations, as evidenced by their intrinsic viscosity behavior. Additionally, it was observed that the dendrimers with a bond angle of $\phi =0^{\circ }$ exhibited much shorter relaxation times compared to dendrimers with higher bond angle limitations, such as $\phi =30^{\circ }$ and $\phi =150^{\circ }$. Consequently, the highest values of intrinsic viscosity maxima [16] was observed in dendrimers that impose the most stringent bond orientation limitations, specifically at $\phi =150^{\circ }$. The aforementioned observation elucidates the complex correlation between bond orientation and the intrinsic viscosity characteristics of semiflexible dendrimers.

Lu et al. [53] introduced a new theory for the intrinsic viscosity of polymers with various architectures, from linear chains to dendrimers, based on a partially permeable sphere model. This model incorporates two phenomenological functions, κ(r) and ξ(r), to describe hydrodynamic interactions. The theory combines Debye’s [54] and Einstein’s theories [55], resulting in a simple expression for intrinsic viscosity that avoids complex multi-body interaction calculations. An intrinsic viscosity expression accounting for both Debye and Einstein contributions was obtained. The calculated intrinsic viscosities for linear and star polystyrene in both θ and good solvents closely match experimental data, typically deviating by less than 4%. The theory also predicted that the Mark–Houwink–Sakurada (MHS) exponent for hyperbranched polymers is less than 0.4 and decreases with molecular weight. For dendrimers, it accurately predicts the non-monotonic behavior and the peak in intrinsic viscosity. Thus, the findings highlighted that polymer architecture significantly influences intrinsic viscosity with different behaviors observed for various structures. Furthermore, the authors in the referenced work [16] conducted intrinsic viscosity estimates by manipulating orientation angles while taking into account hydrodynamic interactions as well in their absence and the excluded volume interactions. In subsequent years, an examination was conducted on the rheological properties of model dendrimers in order to observe the distinct peaks in intrinsic viscosity as a function of generation growth. This analysis incorporated the consideration of excluded volume interactions, utilizing the Rouse–Zimm theory as the theoretical framework. Rai et al. [52] conducted a study on the dynamics, and their investigation focused on the relationship between the intrinsic viscosity of dendrimers and their excluded volume parameters, namely $v_{\theta }$ and $v_{\psi }$, as well as their generation. This was compared to dendrimers with the mean-field excluded volume, where the value of $v_{flory}$ was determined using Flory’s theory [56]. The variable $v_{flory}$ displayed a comparable pattern in the intrinsic viscosity of dendrimers. The numerical value roughly corresponds to the result obtained by the geometric technique for the given parameters $\left(v_{\theta }=0.87;v_{\psi }=3.21\right)$. This alignment was in accordance with the geometric methodology employed for the computation of excluded volumes, as outlined within the framework of Flory’s theory [56]. The observation was clear that dendrimers exhibiting different excluded volume parameters displayed the same qualitative pattern, which was characterized by a distinct maximum at specified stages of growth. The observed tendency was in opposition to the previously suggested dendrimer model under conditions of favorable solvent [36], where they conducted a study on conformational properties. Nevertheless, these findings are consistent with facts derived from a range of experimental, modeling [27,57], and theoretical investigations [16]. The intrinsic viscosity is influenced by the precise values of the excluded volume parameters. Dendrimers characterized by lower values of $v_{\theta }$ and $v_{\psi }$ demonstrated elevated inherent viscosity across all generations with the exception of the initial generation. A significant peak in intrinsic viscosity was seen at the sixth generation when the excluded volume parameters were set to low values ($v_{\theta }=0.13$ and $v_{\psi }=1.75)$. As the strength of excluded volume interactions decreases, the location of the maximum changes toward higher generations. The displacement of the typical maximum has been shown in previous theoretical [16], computational [7], and experimental [7] investigations as well. Figure 5 depicts graphically the comparisons between different analytical models (Figure 5a,b), an analytical model with experimental results (Figure 5c), and an analytical model with simulation results (Figure 5d).

Interestingly, in contrast to the common misconception that dendrimers have a dense core and open periphery, a careful explanation [58] between the molecular weight and volume of the dendrimer for estimating intrinsic viscosity was discovered and displayed the current picture of dendrimers as a transition from a non-compact sphere to a dense space-filling hyperbolic topology [58]. The exceptional maximum in the generation dependent intrinsic viscosity was examined [58]. A cross-over from Euclidean to non-Euclidean dimensions marks the beginning of this structural transition, which was marked by the position of this maximum. The topology of dendrimers is characterized by this structural transition. In three dimensions, the real accessible volume is not a continuous cubic power-law but a discontinuous function of generation. This suggests that the terminal groups are dispersed throughout the core and periphery of dendrimers, whereas the core is dense. Due to their enormous density and densely packed peripheries, huge dendrimers demonstrate a fractal nature with fractal dimensions larger than 3 (refer to the highlighted equation in Figure 5). As a result, it was discovered that dendrimers have compact cores and dispersed terminal groups in both the core and periphery. Large dendrimers therefore display a fractal nature with a fractal dimension greater than 3, as they are extremely dense entities with crowded peripheries. A maximum in the [η] plot is produced by such a shift in shape, which is followed by a negative slope. The value of α, which here refers to the exponent factor from the Mark–Houwink–Kuhn–Sakurada (MHKS) equation, changed from being positive for lower generations to being negative after a specific value of [η] corresponding to a threshold generation. Therefore, as dendrimers develop through generations, the location of the peak in intrinsic viscosity denotes the beginning of a structural change. This shift is accompanied by an internal reorganization of the dendrimer’s component monomers, which results in a change in the dendrimer’s overall shape.

The viscoelastic qualities are a subset of rheological properties that focus on the materials’ combined viscous and elastic behavior. Relaxation moduli, specifically the storage modulus $G^{\prime }\left(\omega \right)$ and the loss modulus $G^{\prime \prime }\left(\omega \right)$, are key factors in understanding dendrimer viscoelasticity. The storage modulus $G^{\prime }\left(\omega \right)$ measures the elastic energy stored in a material, indicating its stiffness and capacity to return to its original shape after deformation. In contrast, the loss modulus $G^{\prime \prime }\left(\omega \right)$ measures the viscous energy dissipated as heat, indicating the material’s damping and flow characteristics. These moduli are especially important in polymer research because they help characterize the mechanical properties and relaxation dynamics of complicated structures like dendrimers. These moduli are measured using rheological techniques, which provide a full understanding of the material’s relaxation spectrum across a range of frequencies. Theoretical models, such as the extended Gaussian structure model, are used to understand how molecular architecture and topology affect polymer mechanical characteristics and relaxation behavior. Table 1 highlights studies undertaken to determine various relaxation moduli, such as the storage modulus, mechanical relaxation modulus, and shear stress relaxation modulus, including their time and frequency dependency.

### 3.2. Conformational Behavior of Model Dendrimers

Conformational transitions in dendrimers pertain to alterations in the spatial configuration and morphology of dendrimer molecules in reaction to external stimuli or environmental circumstances. The comprehension of conformational transitions has significant importance in the customization of dendrimer characteristics to suit certain applications. The transitions observed in the dendrimers can be characterized by changes in their flexibility, compactness, and overall form. The integration of theoretical and computational inquiries with experimental examination of macromolecules facilitates the comprehension of the relationship between their molecular structure and conformational attributes in a solvent environment. Scattering experiments [64,65] and computer simulations [66,67,68,69] play a crucial role in the examination of the morphology and internal composition of dendrimers across different domains. The determination of the static structural factor S(q) of dendrimers in a solution is carried out in scattering experiments. Valuable information, such as the pair correlation function and the segment density profile, can be derived from the provided data by employing techniques such as inverse Fourier transform [57,67,68] and square root deconvolution [64,65]. In contrast, computer simulations provide a means to directly assess the radial density profile and pair correlaetion function, enabling the subsequent determination of S(q) using Fourier transformation. So, both experimental and simulation methodologies offer valuable insights into important parameters such as the radius of gyration $\left(R_{g}\right)$ and hydrodynamic radius $\left(R_{h}\right)$ by directly analyzing the pair correlation function. Moreover, the shape factor, denoted as (ρ), which is the ratio of $R_{g}$ to $R_{h}$, is a significant parameter that provides insights into the morphology, dimensions, and organization of dendrimers and other particle systems.

The radius of gyration $R_{g}$ is an important metric to calculate first when researching dendrimer conformations since it offers a basic assessment of the dendrimer’s overall size and spatial extent. Meanwhile, features such as the shape factor, asphericity, and branching density provide extensive information on the dendrimer’s structure. This fundamental understanding of the dendrimer’s size and density distribution is required for comparing different generations, studying solvent interactions, and determining physical attributes. Determining $R_{g}$ initially offers the framework for a thorough examination of more complex structural characteristics. Table 2 below summarizes major discoveries on $R_{g}$ for several types of dendrimers.

A metric that depicts how the complexity of a fractal pattern increases with scale is referred to as the fractal dimension, which is denoted by the symbol $D_{f}$. In many cases, it results in non-integer values that encapsulate the pattern’s intricacy and space-filling features. It does this by quantifying how the detail or density of a fractal changes as the observation scale changes. A more critical description of natural and irregular shapes is provided by the fractal dimension, in contrast to the usual dimensions, which are integers (for example, 1D for lines and 2D for planes).

The relationship between the radius of gyration $R_{g}$ and the fractal dimension $D_{f}$ is

(10) $$M\propto {\left(\frac{R_{g}}{a}\right)}^{D_{f}}$$

Here, the aggregate mass is denoted by *M*, the radius of gyration is denoted by $R_{g}$, the radius of the single constituent particle (monomer) is denoted by *a*, and the fractal distance is denoted by $D_{f}$. Although some general trends have been reported for the fractal dimension $\left(D_{f}\right)$ of dendrimers, Table 3 shows that lower fractal dimensions, around 1.67, lead to more open and less compact structures. A magnitude between 2.4 and 2.8 is a moderate level of compactness, and fractal dimensions closer to 3.0 are associated with highly compact, space-filling dendrimers. We discuss in the later half of this section that the fractal dimension ($D_{f}$) of semiflexible dendrimers depends on their conformation and bond orientation angle (ϕ). For compressed conformations (ϕ between 0 and π/2), $D_{f}$ is nearly constant at about 4, indicating compact structures. For extended conformations (ϕ between π/2 and π), $D_{f}$ decreases as ϕ increases, ranging from 2.00 to 1.63, suggesting less space-filling structures. Higher generation dendrimers exhibit fractal dimensions greater than 4 due to their highly branching topology. Thus, the findings highlight the role of ϕ in determining dendrimer conformations and interactions. Some authors [52] claimed that a Euclidean to non-Euclidean conformation transition possibly causes $D_{f}$ > 3.

Burchard [76] and Hearst [77] have conducted studies on static phenomena and sedimentation, respectively. Kleppinger et al. [78] used small angle X-ray scattering (SAXS) to experimentally analyze highly diluted solutions of poly(benzyl ether) dendrimers, and the results provide important insights into the structural properties of individual molecules. Ramzi et al. [79] aimed to investigate the structural characteristics and intermolecular interactions of amine-terminated poly(propylene imine) dendrimers in solution using small-angle neutron scattering (SANS). The study examines how dendrimer concentration and acidity influence these interactions, showing that the intermolecular interactions between dendrimers increase with concentration, leading to a pronounced interference peak. Meanwhile, the investigation [80] regarding the structural characteristics of poly(propyleneimine) dendrimers up to the fifth generation employing SANS, viscosimetry, and molecular dynamics simulations to determine the dimensions and density distributions in various solvents concluded that PPI dendrimers exhibit a compact, space-filling structure with a fractal dimensionality of approximately 3. Ballauff [81] analyzed the structure of a fifth-generation dendrimer in solution using SANS. The analysis effectively elucidates the radial scattering length density and highlights the strong structural fluctuations within the dendrimer. Other studies featured the use of SANS to understand how counterions and the protonation of amino groups affect the dendrimer’s molecular conformation and interactions [82], understanding how dendrimer generation affects counterion association and molecular conformation under varying acidity conditions [83] and how molecular protonation and dendrimer concentration affect the dendrimer’s radial density profile and overall conformation using SANS [84].

The study of scattering patterns at lower concentrations primarily reveals form factor contributions from individual molecules. In this context, form factors refer to the scattering profiles attributable to the shape and size of single particles without considering interactions between them. As the concentration of particles increases, a notable decrease in forward scattering intensity is observed, which indicates the onset of significant intermolecular interactions. These interactions disrupt the scattering pattern predicted solely by form factors and need to be considered in the analysis. In dilute solutions, the Guinier approximation is effective for determining the radius of gyration ($R_{g}$), a measure of the overall size of the particles, up to a certain *q*-value (where *q* is the scattering vector). The Guinier plot, which is a plot of ln(I(q)) versus $q^{2}$, where I(q) is the scattering intensity, remains linear at low *q*-values, allowing for the accurate determination of $R_{g}$. However, at higher concentrations, the Guinier approximation fails due to the increased influence of intermolecular interactions, leading to deviations from linearity in the Guinier plot. To understand how particle size changes with concentration, the radius of gyration ($R_{g}$) was determined. This measure provides insight into the spatial dimensions of the molecules in solution and how they are affected by concentration. The Kratky plot, which plots $I\left(q\right)q^{2}$ versus *q*, is useful for analyzing the shape of the particles. In this study, the Kratky plot for dendrimer complexes displayed a maximum, which is indicative of a transition to a more compact, globular shape for the dissolved molecules. This finding was pivotal in identifying the structural characteristics of dendrimer molecules, as a peak in the Kratky plot typically signifies a globular structure. In comparison, Topp et al. [85] conducted an extensive study using SANS and X-ray scattering to examine changes in dendrimer size in concentrated solutions. Their work revealed a distinctive peak in the Kratky plot, which is characteristic of particles with a relatively high internal segment density. Using SANS, Topp et al. [85] systematically examined the average dimensions of poly(amido amine) (PAMAM) dendrimers under various conditions, including temperature fluctuations, mixture compositions of good solvents and non-solvents, and different solvent environments. Their findings indicated that the size and shape of PAMAM dendrimers are highly sensitive to the surrounding environment, providing detailed insights into their structural behavior in solution. Similarly, Chen et al. [86] investigated the behavior of dendrimers in coatings using small-angle scattering techniques. Their study focused on understanding how dendrimers interact within a coating matrix and how these interactions affect the overall properties of the coating. They observed that dendrimers tend to form compact structures at higher concentrations, which is similar to the findings of the Kratky plot analysis in the present study. Their work underscored the importance of considering concentration effects and intermolecular interactions when analyzing the structural properties of dendrimers in various applications.

**Figure 6:**
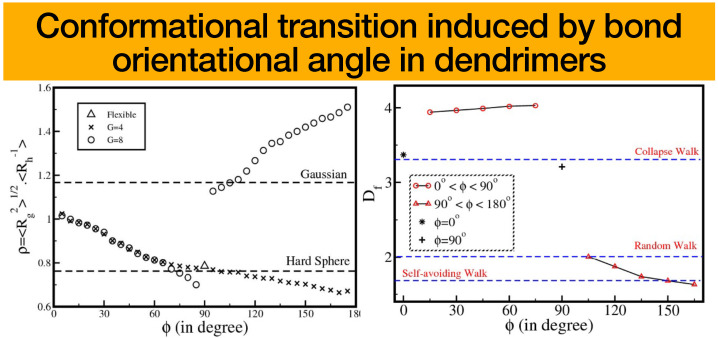
The presented figure illustrates the conformational shift in dendrimers with semiflexibility. It showcases the change in shape factor (ρ) and fractal dimension ($D_{f}$) in relation to the degree of semiflexibility. Reprinted from [18] with the permission of AIP Publishing and for the reproduction of material from Soft Matter, [19], which was published by the Soft Matter Owner Societies.

In the study conducted by Kumar and Biswas [18], they examined the impact of several environmental factors on the conformational characteristics and morphology of dendrimers. The researchers employed various methodologies, including scattering experiments and computer simulations, in order to obtain insights on the behavior of dendrimers. The presented study investigated the relationship between the shape factor, denoted as (ρ), and the bond orientation angle, referred to as ϕ, in the context of semiflexible dendrimers at generations 4 and 8. It is worth mentioning that in compressed conformations where the angle (ϕ) is less than π/2, the parameter (ρ) exhibited a decreasing trend as the angle (ϕ) grows regardless of whether it pertains to a lower or higher generation. In contrast, it has been shown that for extended conformations where the bond orientation angle (ϕ) is more than π/2, and for higher-generation semiflexible dendrimers with a generation value of 8 (G=8), there is an increase in the shape factor, (ρ), as the bond orientation angle (ϕ) increases. In the case of lower generation expanded conformations, there is a drop in the value of ρ as the value of ϕ grows. These observations were consistent with experimental results, which demonstrate that the quality of the solvent has an impact on the values of (ρ). Moreover, the findings indicate that when the variable ϕ grows, the shape factor tends to approximate the limit of a hard sphere for compressed conformations (where 0<ϕ<π/2), and it shifts toward more open structures for extended conformations (where π/2<ϕ<π), as illustrated in Figure 6 (left).

In addition, the investigation also examined the potential influence of factors such as the bond direction angle θ, generation *G*, and functionality *f* on the conformational transition’s dependence on ϕ. It was observed that the parameter ρ exhibits a linear increase with respect to the variable θ across all three conformations. However, this relationship does not suggest any notable structural transition. This observation implies that for a given value of ϕ, the configurations of dendrimers do not undergo significant alterations as a result of variations in θ. The thermodynamic validation of the substantial conformational transition in dendrimers, which is dependent on variations in the shape factor (ρ) and the bond orientation angle ϕ, was achieved through an examination of the configurational free energy. Subsequently, it became apparent that the configurational free energy exhibits an upward trend as the angle θ grows in dendrimers possessing a bond orientation angle of $\phi =150^{\circ }$, but it remains rather stable for dendrimers with bond orientation angles of $\phi =0^{\circ }$ and $\phi =30^{\circ }$. The variation of free energy with respect to θ is continuous for a certain value of ϕ, where $\phi =0^{\circ }$ is situated between the compressed state at $\phi =30^{\circ }$ and the expanded state at $\phi =150^{\circ }$.

In order to advance their research regarding semiflexibility [18], their work closes the gap between earlier models and dendrimers with excluded volume interactions, offering insightful knowledge into the structural adaptability of semiflexible dendrimers by successfully identifying the fractal dimension of semiflexible dendrimers [19]. There are three main scaling regimes that are typical of polymers with various levels of branch flexibility at a given generation, and the fractal dimension ranges widely. The study showed that either adjusting the hierarchy of possible conformations at a given generation or reducing the number of generations during growth can be used to precisely control the internal structure of dendrimers. Any macromolecule’s fractal dimension $\left(D_{f}\right)$ can be calculated using the scaling equation between the number of monomers (N) and the radius of gyration $\left(R_{g}\right)$. Experimental and theoretical studies show discrepancies in the reported values of $D_{f}$. For instance, Mallamace et al. experimentally found that $D_{f}>4$ for higher-generation dendrimers, while molecular dynamics simulations reported $D_{f}\approx 3$. This article reconciles these differences by tuning the bond orientation angle ϕ within the optimized Rouse–Zimm model framework. They determined the fractal dimension of flexible dendrimers using observed scattering patterns. The discussion centering on the effects of bond orientation angles on higher generation dendrimer fractal dimensions was held by the group. They pointed out that in earlier experimental and theoretical studies, there have been inconsistencies in the reported values of fractal dimensions. The fractal dimension is nearly constant at about 4 for compressed conformations of semiflexible dendrimers (ϕ is between 0 and π/2). This is consistent with the results of simulations of dendrimers of higher generations. In contrast, the fractal dimensions for extended conformations (where ϕ is between π/2 and π) fall as ϕ increases and can range between 2.00 and 1.63. As ϕ rises in the extended conformation regime, semiflexible dendrimers become less space-filling structures, with values that are similar to those of the random walk and self-avoiding walk models of linear polymers. Overall, they emphasized the importance of ϕ in capturing the physical characteristics of various dendrimer conformations and their resemblance to interactions between polymers and solvents; see Figure 6 (right). They also pointed out that compressed conformations of lower generation dendrimers display behaviors ranging from a random walk to extremely compact globular forms. These compressed conformations surpass the proportions of a compact collapsed walk as generations progress and grow much denser. Particularly at higher generations, the highly branching topology of dendrimers produces a pseudo-fractal structure with a fractal dimension greater than 4, which denotes a very uneven surface. The results of this work demonstrate that the parameter ϕ may be used to predict the degree of polymer–solvent and polymer–polymer interactions precisely, enabling the investigation of a variety of dendrimer conformations.

### 3.3. Relaxation Dynamics of Model Dendrimer

Dendrimer’s relaxation moduli act as windows into their environment, allowing us to observe how these intricate molecules move and interact. They provide information on how rigid, flexible, or semiflexible dendrimers respond to stress as well as how they manage changes over time. Semiflexibility has a noticeable impact on the normal modes as seen in some relaxation moduli examples, such as $G^{\prime \prime }\left(\omega \right)$ and $\Delta \epsilon^{\prime \prime }\left(\omega \right)$. The phenomenon of dielectric relaxation is a fundamental observation that occurs in dendrimers and other materials when they are exposed to an external electric field [87]. In the study of dendrimers, a comprehensive comprehension of dielectric relaxation is necessary for the purpose of characterizing their electrical characteristics and dynamic behavior. The dielectric constant of dendrimers is characterized by two components: the real component, denoted as $\epsilon^{\prime }$, and the imaginary component, which is denoted as $\epsilon^{\prime \prime }$. The real component, $\epsilon^{\prime }\left(\omega \right)$, is a critical parameter that quantifies the ability of dendrimers to undergo polarization and store energy in the form of electric dipoles. Similarly, the imaginary component, $\epsilon^{\prime \prime }$, holds equal significance in dendrimers. The measurement pertains to the efficacy of dendrimers in dissipating energy, primarily in the form of heat, due to molecular reorientations, electronic transitions, or other relaxing processes taking place inside the dendritic structure. A greater value of $\epsilon^{\prime \prime }$ indicates a more significant level of energy dissipation. Both $\epsilon^{\prime }\left(\omega \right)$ and $\epsilon^{\prime \prime }\left(\omega \right)$ exhibit frequency dependence, and their changes with respect to frequency provide valuable information. The utilization of frequency dependency enables the comprehension of the dispersion of relaxation durations inside dendrimers.

**Figure 7:**
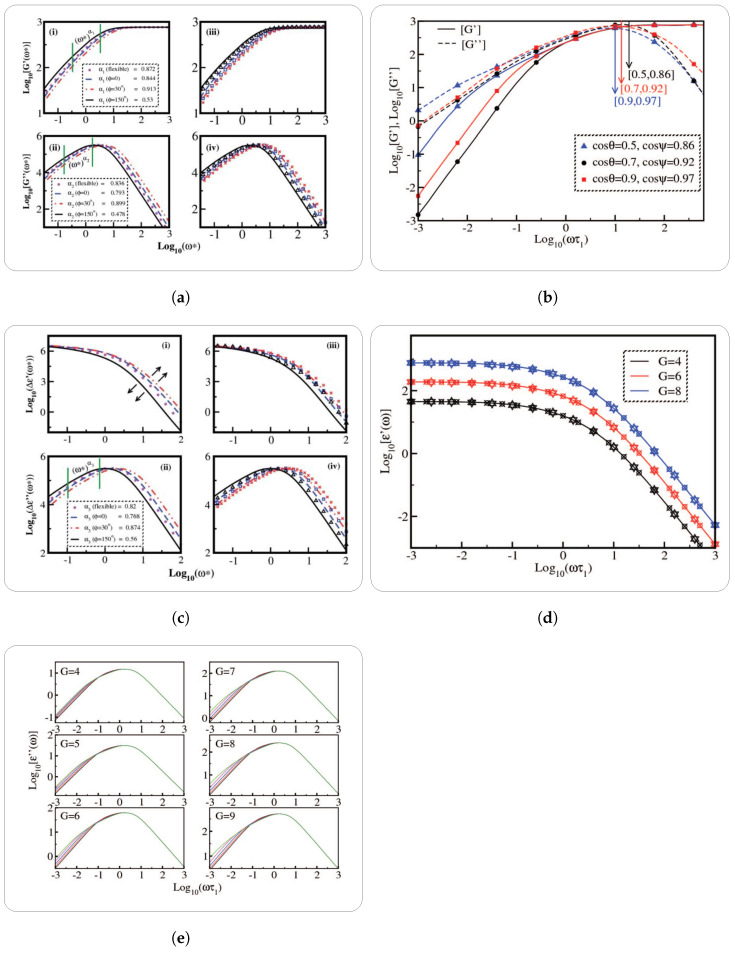
The figure illustrates the trend of mechanical mechanical relaxation moduli of dendrimers (**a**) as a function of semiflexibility and (**b**) as a function of excluded volume parameters. (**c**) The dielectric relaxation moduli of dendrimers are displayed for model dendrimers with semiflexiblity and exlcuded volume parameters, while real and imaginary part of dielectric relaxation moduli are displayed in plots (**d**,**e**), respectively. Also, (**d**) displayed logarithmic plot of the real part of the dielectric relaxation modulus, (ω) for G = 4, 6, 8 dendrimers with varying excluded volume parameters. Here, line, circles, up-triangles, down-triangles, and crosses denote dendrimers with values of the excluded volume parameters $v_{\theta }$, $v_{\phi }$ as tabulated in ref. [52], from top to bottom, respectively.Reprinted from [17,38] with the permission of AIP Publishing. (**a**) Reprinted from [17] with the permission of AIP Publishing. (**b**) Reprinted from [52] with the permission of AIP Publishing. (**c**) Reprinted from [17] with the permission of AIP Publishing. (**d**) Reprinted from [52] with the permission of AIP Publishing. (**e**) Reprinted from [52] with the permission of AIP Publishing.

The investigation of dielectric susceptibility components in the research unveiled fascinating patterns within semiflexible dendrimers across different bond orientation angles and generations [17]. For lower frequencies, the magnitudes of these components were equal across all bond orientations and generations. However, they showed a rise that was dependent on frequency and proportional to the inverse square of the normalized frequency, ${\left(\omega^{*}\right)}^{-2}$. In the region of high frequency, the decay of these entities followed an inverse relationship with respect to $\left(\omega^{*}\right)$, which was subsequent to the attainment of local maxima. This pattern was particularly evident for generations exceeding four unless the entities were in expanded conformations. In the latter scenario, the decay occurred at a faster rate, which was characterized by a power law exponent of about −1.3. The displacement of distinctive peaks moved toward higher frequencies as the generation increases, resulting in an expansion of the susceptibility’s dynamic range (Figure 7a). It is worth mentioning that the peak values observed in the semiflexible dendrimer with an angle of $\phi =0^{\circ }$ coincided with those of $\phi =30^{\circ }$. Conversely, for $\phi =150^{\circ }$, the peak values were observed at lower frequencies in comparison to the models with $\phi =0^{\circ }$. Thus, the aforementioned results offered significant contributions to the understanding of the dynamic characteristics of semiflexible dendrimers across different circumstances, elucidating their relaxation properties and frequency-dependent reactions.

Moreover, the behavior of the real portion of the dielectric susceptibility, designated as $\Delta \epsilon^{\prime }\left(\omega \right)$, in the context of dendrimers with variable excluded volume parameters and dendrimer generations was covered by the authors in another study [38]. For dendrimers with various properties, they displayed their findings through a double logarithmic plot of $\Delta \epsilon^{\prime }\left(\omega \right)$ against normalized frequency $\left(\omega \tau_{1}\right)$. Surprisingly, their findings showed that the magnitude of $\Delta \epsilon^{\prime }\left(\omega \right)$ remained constant despite changes in the excluded volume parameters, indicating that excluded volume interactions between adjacent non-bonded monomers within the dendrimer structure do not affect the dielectric susceptibility (Figure 7b). Furthermore, they observed that the dynamical range of $\Delta \epsilon^{\prime }\left(\omega \right)$ values widens with increased dendrimer generations, indicating a larger frequency dependency. In contrast to earlier theoretical predictions, $\Delta \epsilon^{\prime }\left(\omega \right)$ displayed a power-law decrease in the high-frequency band with an exponent of roughly −1.85 [88]. The authors hypothesized that this discrepancy might be caused by how eigenvalues and eigenvectors interact throughout the mathematical operations. Intriguingly, they discovered that the expanded conformations of semiflexible dendrimers had power-law exponents that mimic those of dendrimers with excluded volume interactions, indicating that the latter accurately simulates the effect of excluded volume interactions in dendrimers. So, the complex dielectric behavior of dendrimers and how they respond to various structural features were clarified by these studies. For the imaginary part, they found that for all generations and excluded volume parameters, the numerical values of $\Delta \epsilon^{\prime \prime }\left(\omega \right)$ increased proportionally with normalized frequency in the low-frequency region, following a power law of about ${\left(\omega \tau_{1}\right)}^{2.13}$. It is interesting to note that the magnitudes in this location showed a substantial relationship with the parameters that were excluded from the volume calculations with magnitudes falling as the parameters were increased. The behavior of $\Delta \epsilon^{\prime }\left(\omega \right)$ in the high-frequency zone, however, was constant for all excluded volume factors with all curves overlapping. The values of $\Delta \epsilon^{\prime \prime }\left(\omega \right)$ in this high-frequency regime declined with normalized frequency as ${\left(\omega \tau_{1}\right)}^{1.33}$ with local maxima in the intermediate frequency range occurring before this for all generations. This behavior in the low- and high-frequency regimes remarkably resembled expanded semiflexible dendrimer conformations. Additionally, the authors observed a cross-over in $\Delta \epsilon^{\prime \prime }\left(\omega \right)$ with different excluded volume parameters, showing that numerical values are higher for larger excluded volume parameters in the intermediate regime (Figure 7c).

**Figure 8:**
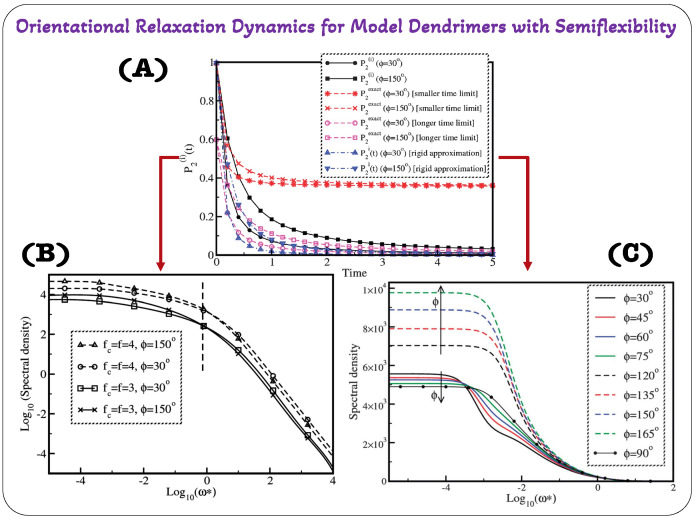
(**A**) The comparison plot of the structural average of $P_{2}^{\left(i\right)}\left(t\right)$ with $P_{2}^{exact}\left(t\right)$ (obtained through an earlier study [92]) for compressed $\left(\phi =30^{\circ }\right)$ and expanded $\left(\phi =150^{\circ }\right)$ conformations of semiflexible dendrimers with branch-point functionality $f_{c}=3,f=3$ and generation G=8. (**B**) Double logarithmic plot of spectral density, $J(\omega^{*})$, as a function of a dimensional frequency, $\omega^{*}$, for compressed and expanded conformations of G=8 semiflexible dendrimers with branch-point functionality $f_{c}=3,f=3$ and $f_{c}=4,f=4$. (**C**) Semi-logarithmic plot of spectral density, $J(\omega^{*})$, as a function of a dimensional frequency, $\omega^{*}$, for G=8 semiflexible dendrimers with branch-point functionality $f_{c}=3,f=3$ for the entire range of bond orientation angle, ϕ(0<ϕ<π). For reproduction of material from PCCP, see [28], which was published by the PCCP Owner Societies.

Dendrimers also display orientational relaxation dynamics, which is different from dielectric relaxation. The ability of dendrimers or segments to change their orientation or alignment in response to environmental factors is directly related to this phenomenon. It entails reorganization in order to achieve a more thermodynamically advantageous orientation. Researchers frequently use NMR analytical theories, experimental approaches [89], and computer simulations [90,91] to analyze orientational relaxation dynamics. These studies offer insightful information about the processes, mechanisms, and variables affecting dendrimer reorientation. The orientational relaxation dynamics of semiflexible dendrimers was carried out inside the optimized Rouse–Zimm formalism in a novel theoretical framework created by Kumar and Biswas [28]. By using a graph theory approach to provide precise limits on the direction and orientation of the individual bond vectors within the dendrimer structure; the concept of semiflexibility was introduced in this analytical model. The preaveraged Oseen tensor was also used to represent hydrodynamic interactions, which are crucial to the behavior of the dendrimer. The analysis of two crucial parameters—the temporal autocorrelation function, designated as $M_{1}^{\left(i\right)}\left(t\right)$, and the second-order orientational autocorrelation function, denoted as $P_{2}^{\left(i\right)}\left(t\right)$, coupled with its cosine Fourier transform, spectral density J(ω), were the main emphasis of the study. Two important variables were examined in relation to these functions: the branch-point functionality and the level of semiflexibility. The innovative way that Kumar and Biswas calculated $M_{1}^{\left(i\right)}\left(t\right)$ set their methodology apart from earlier research by Perico and Guenza [92,93]. (For a comprehensive understanding of the formalism used in this study, readers are referred to the referenced article [28].) Their research addressed an issue where the equation for $M_{1}^{\left(i\right)}\left(t\right)$ did not adequately distinguish between flexible and semiflexible dendrimers. Figure 8A illustrates the contrast between these two analytical models through orientational autocorrelation function $P_{2}\left(t\right)$ [28,92].

The article [28] also discusses the evaluation of the local orientational mobility of dendrimers through the utilization of the spectral density function J(ω), which is obtained by performing a cosine Fourier transform of $P_{2}^{\left(i\right)}\left(t\right)$. The authors introduced an approximation technique for computing these functions at both short and long time scales. Furthermore, they compared the accuracy of these approximations with exact expressions for specific scenarios, as documented in the works of Perico and Guenza [92,93].

Observations were made regarding the behavior of the second rank orientational autocorrelation function, $P_{2}^{\left(i\right)}\left(t\right)$ (Figure 8A), for compressed and expanded conformations of semiflexible dendrimers at both small and large times. In this context, $P_{2}^{exact}\left(t\right)$ showed distinct patterns for these two conformation types. The curves of $P_{2}^{exact}\left(t\right)$ gradually approached zero as time extends to infinity, indicating that at large times, the orientation within the dendrimers becomes increasingly random, suggesting isotropic behavior. At small times, however, both compressed and expanded conformations exhibited similar behavior, rapidly relaxing from an initial value of 1. Interestingly, at large times, $P_{2}^{exact}\left(t\right)$ relaxes from a finite value; specifically, it approaches 0.6 for both compressed and expanded conformations of semiflexible dendrimers. Upon closer examination, it becomes evident that the values of $P_{2}^{\left(i\right)}\left(t\right)$ obtained from the theories of Kumar [19] and Perico [92] align with the behavior of $P_{2}^{\left(i\right)}\left(t\right)$ [19]. Particularly, $P_{2}^{\left(i\right)}\left(t\right)$ also diminishes to zero at extended times for both compressed and expanded conformations of semiflexible dendrimers, signifying randomness in an isotropic system. This reduction to zero happens instantaneously for all times except at t=0, where $P_{2}^{\left(i\right)}\left(0\right)$ equals 1, indicating that $P_{2}^{\left(i\right)}\left(t\right)$ values obtained from the theories of Kumar [19] and Perico [92] share qualitative similarity with the behavior of $P_{2}^{exact}\left(t\right)$. Examining the numerical values of $P_{2}^{exact}\left(t\right)$ for compressed and expanded conformations of semiflexible dendrimers, it became evident that at small times, these values closely resemble those derived from equation obtained from the works of [92,93]. However, for all other time intervals, the magnitudes of $P_{2}^{exact}\left(t\right)$ fall between the values of $P_{2}^{\left(i\right)}\left(t\right)$ obtained from the equations in [28,92] for both conformations of semiflexible dendrimers. Notably, the numerical magnitudes of $P_{2}^{\left(i\right)}\left(t\right)$ calculated from Equation [92] consistently surpass those computed within the rigid approximation [28,92] across the entire time range for both compressed and expanded conformations of semiflexible dendrimers. This discrepancy in numerical magnitudes of $P_{2}^{\left(i\right)}\left(t\right)$ was particularly pronounced in the intermediate time zone, where $P_{2}^{\left(i\right)}\left(t\right)$ values derived from the graphical theory approach [28] significantly exceeded those obtained from the rigid approximation for both compressed and expanded conformations of semiflexible dendrimers. Equations (11) and (12) are provided for readers to better comprehend and correlate the expressions for $P_{2}^{exact}\left(t\right)$ and $P_{2}^{\left(i\right)}\left(t\right)$.

(11) $$P_{2}^{exact}\left(t\right)=1-3\left[x^{2}-\frac{\pi }{2}x^{3}\left(1-\frac{2}{\pi }\arctan x\right)\right]$$

(12) $$P_{2}^{\left(i\right)}\left(t\right)=2\times \sum_{k=2}^{N}\frac{{\left({\left(Q^{T}GG^{T}Q\right)}_{kk}^{\left(i\right)}\right)}^{2}}{\mu_{k}^{2}}\exp\left[-2\sigma \lambda_{k}t\right]≃{\left[M_{1}^{\left(i\right)}\left(t\right)\right]}^{2}$$

The spectral density, denoted as J(ω), derived from the cosine Fourier transformation of the second-order orientation autocorrelation function represented as $P_{2}^{i}\left(t\right)$, was also derived in the studies of Kumar [28]. As depicted in Figure 8B, they have presented a double logarithmic plot showcasing the normalized spectral density, labeled as $J(\omega^{*})$, in relation to the reduced frequency, $\omega^{*}$. The investigation centered on two distinct configurations of semiflexible dendrimers, specifically those with G=8, encompassing the compressed $\left(\phi =30^{\circ }\right)$ and expanded $\left(\phi =150^{\circ }\right)$ forms. Additionally, they considered variations in branch-point functionalities, which were denoted as $f_{c}$. Within the low-frequency spectrum, they observed an intriguing pattern wherein the magnitude of the spectral density remains constant across all dendrimer types regardless of frequency. However, it becomes apparent that the magnitude of J(ω) exhibits a pronounced dependence on both the degree of semiflexibility and the branch-point functionality. To clarify, dendrimers characterized by higher branch-point functionality and increased semiflexibility demonstrate numerically higher values of J(ω). In simpler terms, those with $\left(\phi =150^{\circ }\right)$ and $f_{c}=f=4$ display the highest J(ω) values, while their counterparts with $\left(\phi =30^{\circ }\right)$ and $f_{c}=f=3$ exhibit the lowest values. Transitioning into the high-frequency range reveals a distinct behavior. The spectral density decreased with frequency, following a decay pattern of ${\left(\omega^{*}\right)}^{2}$, which was a phenomenon observed for both the compressed and expanded configurations of semiflexible dendrimers featuring branch-point functionalities of $f_{c}=f=3$ and 4. Notably, an interesting occurrence takes place in the intermediate frequency spectrum, which was marked by the dotted line in Figure 8B. In this range, the spectral density curves for compressed and expanded dendrimers diverge noticeably. Crucially, within this intermediate spectrum, the spectral density values were higher for compressed dendrimers than for their expanded counterparts, owing to the observed cross-over effect. This implies that in shaping the spectral density’s behavior in semiflexible dendrimers, the degree of semiflexibility primarily governs the outcome, while changes in branch-point functionality exert a relatively minor influence.

Figure 8C presents the frequency-dependent behavior of the spectral density in G=8 dendrimers with branch-point functionality $f_{c}=f=3$. Regardless of *f*, the spectral density showed a consistent pattern across the entire frequency range. Notably, a cross-over effect was observed in the intermediate frequency region—specifically in the compressed dendrimer conformations (0≤ϕ≤π/2) compared to the expanded ones (π/2≤ϕ≤π). The characteristic area beneath the spectral density curve strongly correlated with the degree of semiflexibility, which is noteworthy as it is independent of the correlation time. In the expanded conformation zone, the characteristic area is maximized and increases with higher ϕ values. In contrast, for compressed dendrimers, this area decreases with ϕ, which is accompanied by a cross-over in the spectral density in the intermediate frequency region. However, the relative change in the characteristic area was more pronounced in expanded conformations. Lastly, flexible dendrimers (ϕ=π/2) exhibited the smallest characteristic area compared to both compressed and expanded conformations.

### 3.4. Application toward Randomly Hyperbranched Geometries

Randomly hyperbranched structures are a class of polymers characterized by a random and highly branched architecture, which is distinct from the regular branching patterns of dendrimers. They exhibit a high degree of branching, allowing for the incorporation of diverse monomers and leading to amorphous, compact structures with unique physical and chemical properties. They may contain defects and dead ends in their branching structure [94]. These defects result from the more irregular and random nature of the branching process. As a consequence, randomly hyperbranched polymers exhibit a less structured, more complex architecture, making them distinct from other cascade molecules. Due to their peculiar topology-dependent behavior, hyperbranched polymers, which are characterized by their random branching patterns, exhibit equilibrium and relaxation properties that are noticeably different from those of typical dendrimers [30,37,95,96]. These analytical models, which take into account elements like semiflexibility and excluded volume interactions, were extended to examine the behavior of more complex branched macromolecules known as hyperbranched structures, in addition to research concentrating on the dynamic behavior of dendrimers. Furthermore, research involving novel systems proved the validity of these characteristics. Therefore, it was necessary to classify these macromolecules in order to determine which properties are most affected by the structural disorder and which ones are mostly untouched both theoretically and experimentally.

**Figure 9:**
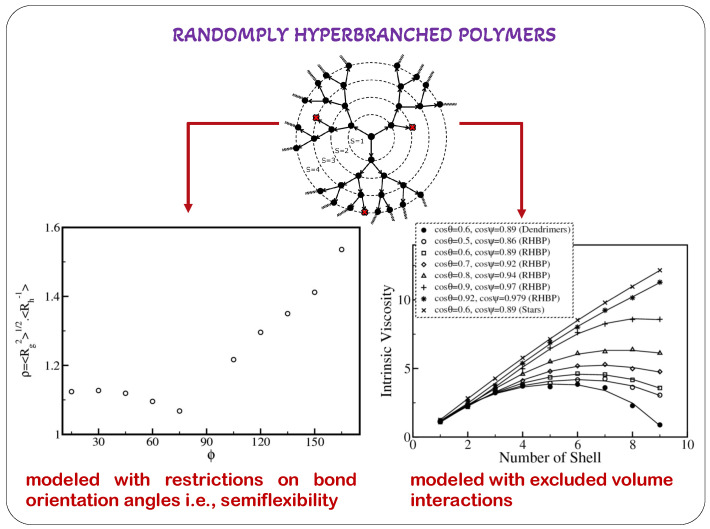
(**Top**): A schematic representation of randomly hyperbranched polymers that have been synthesized using a growth algorithm. Individual beads are used to represent the monomers that make up the polymer, and bond–vector notations are used to indicate the connections that join two monomers. Furthermore, red-cross symbols represent the limit of randomly hyperbranched polymers, beyond which no further growth is possible. Dashed circles represent the concept of a “step” (S). (**Left**): Plot of the variation of shape factor, ρ, for the S=9 semiflexible randomly hyperbranched polymers as a function of bond orientation angle. (**Right**): Intrinsic viscosity of randomly hyperbranched polymers with excluded volume parameters, $\left[v_{\theta },v_{\psi }\right]$, corresponding to different θ and ψ values in the partial draining limit and compared with those of dendrimers and star polymers with same number of monomers with excluded volume parameters, $\left[v_{\theta },v_{\psi }\right]$, corresponding to $\cos_{\theta }=0.6$ and $\cos_{\psi }=0.89$. Reprinted from [97] with the permission of AIP Publishing.

When analyzing the scaling behavior of viscoelastic relaxation moduli with respect to the dimensionless frequency, ω, a clear contrast between randomly hyperbranched polymers and dendrimers becomes obvious. In this environment, dendrimers display a distinctive non-scaling behavior in contrast to randomly branching polymers. In an application of further study, the authors [20] sought to examine the structural and dynamic characteristics of semiflexible randomly hyperbranched polymers within the context of an improved Rouse–Zimm formalism. By placing restrictions on the bond vector’s direction and orientations along with utilizing the preaveraged Oseen tensor to represent hydrodynamic interactions, they added semiflexibility to the model. Understanding how the bond orientation angle ϕ relates to the frequency-dependent relaxation moduli, specifically the storage modulus $\left[G^{\prime }\left(\omega \right)\right]$ and the loss modulus $\left[G^{\prime \prime }\left(\omega \right)\right]$ [20], they looked into this relationship for a certain branching topology and a particular number of growth generations. The shape factor (ρ), configurational free energy (F), static structure factor (S(q)), and scattering intensity (I(q)) were among the structural properties that the authors looked at in order to characterize the molecular conformation of these polymers (Figure 9 (left)).

Examining the intermediate frequency range of the mechanical moduli for both compressed and expanded conformations revealed the peculiar properties of semiflexible randomly hyperbranched polymers. The bond vector’s orientation in this situation brought back the typical power-law scaling behavior at intermediate frequencies. In contrast to prior research [31], where such power-law scaling was absent in semiflexible randomly branched polymers due to their lower disorder, this behavior was akin to the modeling of hyperbranched polymers as Vicsek fractals. Furthermore, it was discovered that both the quantity of monomers and the level of semiflexibility, as determined by the parameter ϕ, affected the extent of the power-law regime in the intermediate frequency range. Additionally, hyperbranched polymers had a noticeable structural change when the parameter ϕ was increased. The expanded conformation’s compactness declined as ϕ rose, whereas the compressed conformation’s compactness stayed largely unaltered. This structural shift was corroborated by the fractal dimensions estimated using Porod’s scaling equation, which changed from $q^{-2.76}$ to $q^{-1.61}$ for the compressed (0<ϕ<π/2) and extended (π/2<ϕ<π) conformations of semiflexible randomly hyperbranched polymers, respectively. In polymers with complicated topologies, the excluded volume interactions were successfully accounted for by this approach [20].

Semiflexibility and excluded volume interactions are very important factors to take into account in the analytical models. We now look at the results of how excluded volume [97] affected the rheology and transport dynamics of randomly hyperbranched polymers after discussing semiflexibility in the previous section. Through the frequency-dependent mechanical moduli $\left[G^{\prime }\left(\omega \right)\right]$ and $\left[G^{\prime \prime }\left(\omega \right)\right]$, rheological properties were evaluated [97]. Meanwhile, the inherent viscosity and self-diffusion coefficient are frequently used to describe the transport dynamics. The study found that collective modes with slower relaxation rates were primarily responsible for mechanical moduli, and that these modes grew stronger as the excluded volume interactions reduced. Higher relaxation rate local modes were unaffected. The mechanical moduli’s intermediate frequency range clearly reflected the internal structure of randomly hyperbranched polymers, which displayed a distinctive power-law behavior that suggested their fractal origin. The excluded volume interactions had a significant impact on the middle frequency. Here, as the excluded volume strength decreased or the quantity of polymer shells increased, the length of the power-law region grew. The strength of the excluded volume interactions was the only factor that determined the precise values of the power-law exponents. Importantly, when the number of monomers crossed a crucial threshold, the randomly hyperbranched polymers exhibited a more broad power-law region than dendrimers.

The intrinsic viscosity of randomly hyperbranched polymers was examined in relation to the number of shells and various excluded volume parameters, $v_{\theta }$ and $v_{\psi }$. These results were compared to dendrimers and star polymers with equivalent monomer counts under the influence of excluded volume interactions (Figure 9 (right)). The plot revealed that as the number of shells increases, the gap between intrinsic viscosity curves of randomly hyperbranched polymers widens for different excluded volume strengths. Notably, the impact of excluded volume interactions is more pronounced in higher shell layers due to an increased number of non-bonded interactions. The intrinsic viscosity’s magnitude grows as excluded volume interactions weaken with maximum and minimum values occurring at specific excluded volume parameters [cosθ=0.92,cosψ=0.97] and [cosθ=0.5, cosψ=0.86], respectively. This demonstrates an inverse relationship between intrinsic viscosity and the strength of excluded volume interactions. Furthermore, the trend of intrinsic viscosity variation with the number of shells falls between that of star polymers and dendrimers with the same monomer count. Intrinsic viscosity increases linearly, similar to star polymers, for very low excluded volume parameters. However, this trend becames predominantly nonlinear with higher excluded volume parameters, plateauing at advanced shell growth. Beyond a certain shell count, intrinsic viscosity reaches a plateau, which slightly dips with further increases in excluded volume interaction parameters, $\left[v_{\theta },v_{\psi }\right]$, corresponding to [cosθ=0.5,cosψ=0.86] and [cosθ=0.6,cosψ=0.89]. This results in a characteristic broad maximum in intrinsic viscosity at intermediate shells, which is akin to dendrimers for stronger excluded volume interactions. Additionally, the existence of a compact, space-filling conformation beyond a critical shell count is noted especially for randomly hyperbranched polymers with larger excluded volume interaction parameters. Conversely, those with smaller excluded volume interaction parameters exhibit voids and resemble linear or star polymers. These findings align with earlier theoretical and experimental studies. Thus, the numerical magnitudes of intrinsic viscosity for randomly hyperbranched polymers fall between those of dendrimers and star polymers, indicating that randomly hyperbranched polymers are relatively more compact than star polymers but less compact than dendrimers with the same monomer count and identical excluded volume interactions. Importantly, this behavior can be fine-tuned by adjusting the strength of excluded volume interactions, which can be achieved experimentally by manipulating solvent conditions. Among the extensive category of hyperbranched polymers, Vicsek fractals hold particular theoretical significance, especially in the company of highly symmetrical entities like dendrimers, which are beyond the scope of this article [98].

## 4. Summary and Perspective

In contrast to continuum chain models such as the worm-like chain, these analytical models conceptualize dendrimers modeled with discrete semiflexiblility and excluded volume. The solution to the matrix form of the Langevin equation can be obtained by utilizing the eigenvectors and eigenvalues, which are made possible by the discrete representation of semiflexible polymers. A wide range of conformational and dynamic factors were investigated in the context of the semiflexibility and topology of dendrimers by the cited authors. These parameters include intrinsic viscosity, diffusion coefficient, mechanical and dielectric moduli, orientational relaxation, radius of gyration, shape factor, and static structure factor, and the utilization of the theory was exemplified by the randomly hyperbranched polymers.

Finally, the analytical modeling of dendrimers and hyperbranched polymers provides crucial insights into their structural behavior, relaxation dynamics, and interaction patterns, all of which are critical for adapting their properties to a wide range of applications. Incorporating characteristics such as hydrodynamic interactions and matrix representations has improved our understanding by allowing for a more complete examination of their unique motions. Semiflexibility has shed information on the influence of bonds on dendrimer geometries by introducing precise limitations on bond orientations. Excluded volume interactions have also proved critical in capturing short-range interactions inside and between dendrimer generations. One noteworthy result is the unique intrinsic viscosity patterns of dendrimers, which peak at specific generations due to intricate mass-volume correlations. Furthermore, the investigation of dielectric relaxation and orientational relaxation dynamics has yielded important information concerning dendrimer viscoelastic characteristics and their responses to environmental variables. Scattering tests and computer simulations have been critical in determining dendrimer morphology, size, and organization, ultimately assisting in dendrimer design and application. The link between form variables and bond orientation angles in semiflexible dendrimers has revealed intriguing tendencies that are consistent with experimental findings, providing vital structural insights. The investigation of fractal dimensions has contributed to a better understanding of dendrimer thermodynamics and physical properties. Overall, this literature review study improves our understanding of dendrimer structural behavior, allowing them to be used in a variety of applications.

When comparing dendrimers to hyperbranched polymers, their complicated branching patterns exhibit different equilibrium and relaxation properties. The addition of semiflexibility and excluded volume interactions to the analytical models used to comprehend these polymers indicated dissimilar behavior in viscoelastic relaxation moduli when compared to dendrimers. Semiflexible randomly hyperbranched polymers exhibit remarkable power-law scaling behavior at intermediate frequencies with variables such as monomer quantity and semiflexibility influencing their features. Excluded volume interactions have a major impact on their rheological and transport properties, resulting in distinct power-law regimes and intrinsic viscosity patterns. In conclusion, through these analytical studies, we have advanced our understanding of dendrimer and hyperbranched polymer behavior, opening the road for their optimal utilization in a variety of disciplines.

## 5. Future Scope

This review represents a comprehensive re-examination of the theoretical propositions put forth by various authors who have focused on investigating the analytical dimensions of elucidating the structural and dynamical attributes of high molecular weight macromolecules, specifically dendrimers and randomly hyperbranched structures. Each constituent, property, and parameter looked over in this review article assumes a pivotal role in comprehending the practical implications of employing these structures in real-world applications like biomacromolecules. The theories and formalisms delineated in the referenced articles within this manuscript exhibit a degree of uniqueness, rendering them highly promising for application to complex polymeric systems encountered in practical scenarios.

## Figures and Tables

**Table 1:** Characteristicsof various time- and frequency-dependent mechanical relaxation moduli.

S. No.	Model	Method	Relaxation Moduli Description	Reference
1.	Poly(propylene imine): derivatives of methyl acrylate (MA) and benzyl acrylate (BA)	Rheometric Scientific Advanced Rheometric Expansion System (ARES) controlled-strain rheometer	The ARES controlled-strain rheometer from Rheometric Scientific: The underivatized dendrimer (G4 PPI) has a greater storage modulus ($G^{\prime }$) than expected by the Rouse model and no liquid terminal behavior, confirming molecular structure-induced elasticity. At low frequencies, $G^{\prime }$ deviates positively from Rouse behavior for the most viscous sample (G5 PPI/BA), indicating additional relaxation modes. G4 PPI’s loss modulus ($G^{\prime \prime }$) has a power law slope of one across all frequencies, characteristic of unentangled polymer melts, while G5 PPI/BA’s is consistently bigger than $G^{\prime }$, exhibiting liquid-like terminal behavior at intermediate frequencies. This suggests that the dendrimer’s branching design and end-group functionalization, rather than entanglements, increase elasticity and shear thinning due to impacts on the intermolecular microstructure.	[59]
2.	(i) Terminal segments (TSs) with attached rigid massive groups and (ii) TSs with lengths different from the inner segments	Rouse model	The adjusted terminal segments affected both the elastic and loss moduli ($G^{\prime }$). High friction of terminal segments increases both $G^{\prime }$ and $G^{\prime \prime }$ in the low-frequency zone. Terminal segment length determines high-frequency moduli. The position of the $G^{\prime \prime }$ maximum, $v_{max}$, is dictated by the terminal segment’s characteristic time, $t_{end}$, which is influenced by friction and length. Generally, $v_{max}$ is linearly dependent on $t_{end}$, indicating that the dendrimer structure and changes greatly impact relaxation processes.	[60]
3.	Semiflexible dendrimer	Rouse–Zimm model	The study’s findings on viscoelastic relaxation moduli, particularly storage and loss moduli ($G^{\prime }$), demonstrate semiflexible dendrimers’ complicated behavior. At low frequencies, the storage modulus, $G^{\prime }$, increases with frequency $\omega^{*}$, indicating a power-law dependence of ${\left(\omega^{*}\right)}^{2}$. Values for $\phi =0^{\circ }$ fall between $\phi =30^{\circ }$ and $\phi =150^{\circ }$ for higher generations, indicating a considerable dependency on bond orientation angle. At high frequencies, $G^{\prime }$ becomes frequency-independent, indicating a plateau behavior. The loss modulus, $G^{\prime \prime }$, rises linearly with $\omega^{*}$ at low frequencies and decays with ${\left(\omega^{*}\right)}^{-1}$ after peaking in the middle frequency range. As angular limitations rise, the maximum values of $G^{\prime \prime }$ move toward higher frequencies dependent on bond orientation angle. These findings imply that bond orientation and generation greatly alter semiflexible dendrimers’ viscoelastic characteristics, affecting relaxation dynamics and mechanical responses under different frequency regimes.	[17]
4.	PPI and PPG dendrimer	Rouse model	In PPG, $G^{\prime }$ displays typical entangled polymer melt characteristics, including terminal relaxation ($G^{\prime }\left(\omega \right)\propto \omega^{2}$) at low and high frequencies, a rubber plateau at intermediate temperatures, and a high-frequency plateau associated with the α process at low temperatures. $G^{\prime \prime }$ shows a primary relaxation peak at low temperatures, a terminal relaxation ($G^{\prime \prime }\left(\omega \right)\propto \omega$) at higher temperatures, and an intermediate power-law regime ($G^{\prime \prime }\left(\omega \right)\propto \omega^{0.6}$) representing Rouse dynamics at lower frequencies. The PPI dendrimer exhibits broad dispersion curves and an intermediate power-law regime ($G^{\prime }\left(\omega \right)\propto \omega^{0.7}$, $G^{\prime \prime }\left(\omega \right)\propto \omega^{0.7}$) at the glass transition temperature (Tg).	[61]
5.	Poly(butyl- carbosilane) dendrimer melts	Molecular dynamic simulations	At low frequencies, $G^{\prime }\left(\omega \right)∼\omega^{2}$ and $G^{\prime \prime }\left(\omega \right)∼\omega$, showing liquid-like terminal relaxation. A scaling exponent of −0.7 for G(t) at high frequencies leads to $G^{\prime }\left(\omega \right)∼G^{\prime \prime }\left(\omega \right)∼\omega^{0.7}$ behavior. The power law in $G^{\prime }\left(\omega \right)$ and $G^{\prime \prime }\left(\omega \right)$ matches experimental data for PPI dendrimer melts with similar molecular weights. Note that $G^{\prime }\left(\omega \right)$ is below $G^{\prime \prime }\left(\omega \right)$ for G2 and G3, while G4’s curves meet at low frequencies before reaching the terminal regime. As generation increases, the terminal $\omega^{1}$ regime of $G^{\prime \prime }\left(\omega \right)$ swings toward lower frequencies, indicating greater macromolecule interaction.	[62]
6.	PAMAM, PPI, and PCS dendrimer melts	Molecular dynamic simulations	The frequency dependences of $G^{\prime }\left(\omega \right)$ and $G^{\prime \prime }\left(\omega \right)$ differ due to the dendrimer chemical structure. PAMAM’s $G^{\prime }\left(\omega \right)$ and $G^{\prime \prime }\left(\omega \right)$ grow quicker than PPI and PCS at low frequencies due to hydrogen bonding entanglements. All dendrimers had mid-frequency slopes of 0.66 and 0.69 for $G^{\prime }\left(\omega \right)$ and $G^{\prime }\left(\omega \right)$, respectively, which aligns with experimental results for similar dendrimers. Interestingly, PAMAM has a region where $G^{\prime }\left(\omega \right)>G^{\prime \prime }\left(\omega \right)$ due to hydrogen bonding, unlike PPI and PCS. Compared to PCS, PAMAM and PPI’s $G^{\prime }\left(\omega \right)$ and $G^{\prime \prime }\left(\omega \right)$ curves behave differently at high frequencies due to their less dense cores and hydrogen bond entanglements. These findings show that hydrogen bonding and dendrimer architecture greatly affect dendrimer melt viscoelasticity.	[63]

**Table 2:** Summary of radius of gyration ($R_{g}$) findings for dendrimers.

Reference	Major Findings on Radius of Gyration ($R_{g}$)
[34]	The value of $R_{g}$ increases with the number of generations, exhibiting distinct scaling tendencies in solvents that are either good or poor. The dense-core and hollow-core models were presented to the audience.
[46]	The fractal nature of dendrimers was brought to light by the fact that the scaling rules for $R_{g}$ were predicted and the relationship between generation number and $R_{g}$ was found.
[66]	The impacts of excluded volume interactions on $R_{g}$ were investigated, and the results demonstrated how steric hindrance influences the total size and shape of dendrimers.
[59]	As the molecular weight grows, the reactivity group ($R_{g}$) increases, and functionalization improves both thermal and mechanical stability.
[70]	As the temperature rises, the $R_{g}$ values fall, which indicates that the structure becomes more compact as the temperature moves higher.
[60]	There is a substantial power-law pattern observed at short periods, and $R_{g}$ appears to rise as the TS length increases.
[61]	There is a considerable relationship between the quality of the solvent and the $R_{g}$ and density profile with inferior solvents resulting in denser structures.
[62]	A linear viscoelastic characteristic is exhibited by $R_{g}$, which scales logarithmically with generation.
[63]	Compared to linear polymers, the $R_{g}$ for higher generations is larger, indicating that the density distribution is more uniform.

**Table 3:** Summary of fractal dimension findings for dendrimers.

Reference	Major Findings on Fractal Dimension $\left(D_{f}\right)$
[71]	According to the study, the aggregation of carboxylate-terminated PAMAM dendrimers with amine-terminated PAMAM dendrimers in aqueous medium results in the formation of fractal structures. Dynamic light scattering (DLS) experiments indicate that diffusion-limited colloidal aggregation (DLCA) is the dominant mechanism, leading to fractal growth with a fractal dimension ($D_{f}$) of approximately 1.67. This fractal aggregation significantly enhances the intrinsic emission intensity of the dendrimers, suggesting that the formation of these complex structures can stabilize excitons and reduce non-radiative pathways.
[72]	Dendrimers exhibit fractal self-similarity, which is characterized by fractal dimensions ranging from 2.4 to 2.8 across dendrimers of different generations. Monte Carlo simulations demonstrated that individual dendrons within an isolated dendrimer spontaneously segregate and are well segregated at equilibrium, which is a result of the dendrimer’s unique architecture. This segregation is observed even in chemically identical dendrons and persists under various generation numbers. The hierarchical organization of dendrimers contributes to their fractal nature, although this self-similarity extends over a relatively narrow range of lengths. The findings suggest that under high-density conditions, such as in poor solvents or at the highest generation numbers, this segregation effect may diminish.
[73]	The dendrimers exhibit fractal properties with fractal dimensions indicating a self-similar structure over a limited range of scales. Monte Carlo simulations revealed that random walks on dendrimer structures show a linear scaling behavior, maintaining a fractal dimension. The results indicated that while finite-size effects influence the observed behavior, the fractal dimension values for dendrimers lie between 2.45 and 2.76, depending on the generation and coordination number.
[74]	The study states that the fractal dimension of star-like poly-ϵ-caprolactone dendrimers increases with both the generation number and the number of segments between branching units. The fractal dimension ranges between the theoretical limits for low-functionality stars (P≈5/3) and high-functionality stars (P≈3), indicating a transition from a loose, polymeric structure to a more compact shape. The results demonstrate that dendrimers with fewer spacer units exhibit more stretched dendrons, preventing a space-filling arrangement, while dendrimers with higher generations and more spacers achieve a more compact, globular shape. These findings provide insights into the conformational behavior of dendrimers in solution under good solvent conditions.
[75]	The PAMAM dendrimers possess a fractal structure with dimensions between 2.0 and 3.0, as determined by both all-atom and coarse-grained simulations. This fractal nature reflects the space-filling capacity and structural complexity of dendrimers.

## Data Availability

No new data were created or analyzed in this study.
